# CD1a promotes systemic manifestations of skin inflammation

**DOI:** 10.1038/s41467-022-35071-1

**Published:** 2022-12-07

**Authors:** Clare S. Hardman, Yi-Ling Chen, Marcin Wegrecki, Soo Weei Ng, Robert Murren, Davinderpreet Mangat, John-Paul Silva, Rebecca Munro, Win Yan Chan, Victoria O’Dowd, Carl Doyle, Prashant Mori, Andy Popplewell, Jamie Rossjohn, Daniel Lightwood, Graham S. Ogg

**Affiliations:** 1 MRC Human Immunology Unit, MRC Weatherall Institute of Molecular Medicine, University of Oxford, Oxford, UK; 2 Infection and Immunity Program and Department of Biochemistry and Molecular Biology, Biomedicine Discovery Institute, Monash University, Clayton, VIC Australia; 3 UCB Pharma, 208 Bath Road, Slough, SL1 3WE UK; 4 Institute of Infection and Immunity, School of Medicine, Cardiff University, Cardiff, CF14 4XN UK

**Keywords:** Inflammation, Cellular immunity, Psoriasis, MHC

## Abstract

Inflammatory skin conditions are increasingly recognised as being associated with systemic inflammation. The mechanisms connecting the cutaneous and systemic disease are not well understood. CD1a is a virtually monomorphic major histocompatibility complex (MHC) class I-like molecule, highly expressed by skin and mucosal Langerhans cells, and presents lipid antigens to T-cells. Here we show an important role for CD1a in linking cutaneous and systemic inflammation in two experimental disease models. In human CD1a transgenic mice, the toll-like receptor (TLR)7 agonist imiquimod induces more pronounced splenomegaly, expansion of the peripheral blood and spleen T cell compartments, and enhanced neutrophil and eosinophil responses compared to the wild-type, accompanied by elevated skin and plasma cytokine levels, including IL-23, IL-1α, IL-1β, MCP-1 and IL-17A. Similar systemic escalation is shown in MC903-induced skin inflammation. The exacerbated inflammation could be counter-acted by CD1a-blocking antibodies, developed and screened in our laboratories. The beneficial effect is epitope dependent, and we further characterise the five best-performing antibodies for their capacity to modulate CD1a-expressing cells and ameliorate CD1a-dependent systemic inflammatory responses. In summary, we show that a therapeutically targetable CD1a-dependent pathway may play a role in the systemic spread of cutaneous inflammation.

## Abstract (web summary)

Skin inflammation is often accompanied by systemic disease, yet the pathways that regulate this escalation are little known. Here authors show that transgenic expression of human CD1a in mice leads to the escalation of experimental skin inflammation and systemic inflammatory disease, and the generalized symptoms could be alleviated by blocking antibodies developed against CD1a.

## Introduction

The immunopathology of inflammatory skin disorders, including psoriasis and atopic dermatitis (AD), combines barrier dysfunction and immune dysregulation. This is exemplified by studies that identified the gene encoding filaggrin as a key genetic determinant of AD^1^, and the efficacy of anti-IL-4Rα, Dupilumab, in AD clinical trials^2^. The skin is more than a superficial line of defence and has recently been suggested to have wide-reaching systemic influence, with skin disease associated with clinical and subclinical systemic inflammation. For example, psoriasis is linked to multiple co-morbidities, including psoriatic arthritis, cardiovascular disease (CVD) and atherosclerosis, obesity and diabetes, inflammatory bowel disease (IBD) and psychological disorders. Classically, AD forms the first step of the atopic march, the progressive development of non-cutaneous atopic disease, including allergic asthma, rhinitis and food allergy, where exposure to allergens via the gut potentially promotes tolerance, yet via the skin can induce allergic sensitization^3,4^. More recently, AD has also been linked to CVD. A number of interesting correlative studies have observed systemic biomarker serum profiles indicative of systemic inflammation in both psoriasis and AD. Notably, one study compared AD and psoriasis sera and a common inflammatory profile was detected. Additionally, distinct biomarkers were identified for psoriasis, including IL-17A, and protein markers of angiogenesis, endothelial activation and lipid metabolism; and moderate/severe AD, including a number of pan T helper subset cytokines and chemokines, and proteins involved in atherosclerosis, tissue remodelling, and angiogenesis^5^. Effector molecules modulated in the blood of patients with skin disease may underlie the dissemination of inflammation, including the progression of atopic diseases and co-morbidities such as CVD^5–10^.

CD1a is a virtually monomorphic major histocompatibility complex (MHC)I-like molecule that is constitutively highly expressed by epidermal Langerhans cells (LC) and can be induced on other skin-infiltrating cells, including dendritic cell (DC) subsets^11,12^, and innate lymphoid cells (ILC)^13^. We and others have shown that CD1a presents lipid antigens to T cells and contributes to psoriatic, allergic and dermatitic inflammation^13–20^. Cross-talk between the skin and other peripheral and immune organs likely underpins many inflammatory disorders; however, mechanisms of this dialogue remain undiscovered. Given the skin-dominant expression of CD1a, most studies have focused on skin-specific functional effects, although we and others have shown the presence of circulating CD1a-reactive T cells^15,18,21,22^. CD1a mRNA has been detected more widely than protein, including in the gut and lungs^23,24^. Indeed, there is evidence CD1a expression may be altered at non-cutaneous sites during disease, including IBD^25,26^. CD1a is known to amplify the imiquimod skin response^18^, but there have been no studies on associated systemic sequelae.

Here, we generate a human CD1a transgenic mouse and human CD1a-reactive T cells and characterize anti-CD1a antibodies using human and mouse assays. The findings confirm CD1a-dependent effects in the skin, but extend to systemic effects, with implications for the treatment of inflammatory diseases.

## Results

### Generation of CD1a transgenic mice

**Fig. 1:**
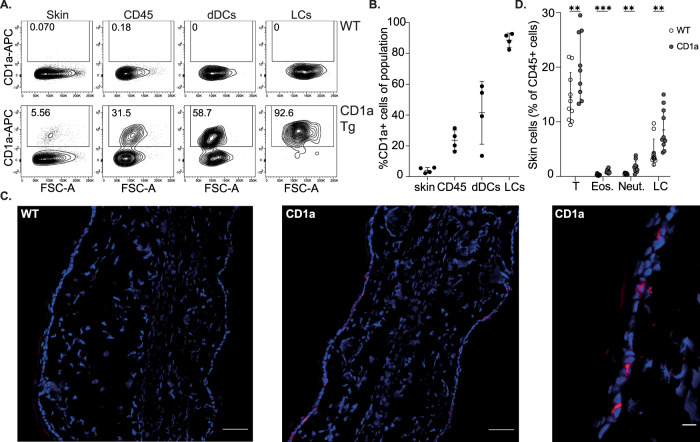
Characterization of CD1a transgenic mouse. **A** Representative flow cytometry plots and **B** graphical summary of CD1a protein expression by wild-type (WT) and CD1a transgenic (CD1a Tg) C57BL/6 J mice. CD1a protein expression evaluated on (left-right) total live ear skin cells, CD45 + skin cells, dermal dendritic cells (dDCs, CD45^+^/CD11c^+^/langerin^-^) and Langerhans cells (LCs, CD45^+^/CD11c^+^/langerin^+^). *n* = 4 for all groups examined over 2 independent experiments. Mean ± SD is shown. n represents biologically independent animals in each group. **C** CD1a protein expression within ear skin of wild-type (WT) and CD1a transgenic (CD1a) mice, visualized by immunofluorescence. Cryosections were stained with DAPI (blue) and anti-CD1a AF-594 (OKT6, red), scale bars left to right 50, 50 and 10 µm. Data shown are representative of 3 independent experiments. **D** Frequency of T cells (T), Eosinophils (Eos.), Neutrophils (Neut.) and Langerhans cells (LC) in the skin of wild-type (WT) and CD1a transgenic (CD1a) mice as measured by flow cytometry as a percentage of CD45 + cells. *n* = 11 examined over 3 independent experiments. Grouped analysis multiple unpaired two-tailed *t*-tests with Holm-Sidak correction, mean ± SD, **P < 0.01; ***P < 0.001. Mean ± SD is shown. n represents biologically independent animals in each group. Exact *p*-values are recorded in Supplementary Table 1. Source Data are provided as a Source Data file.

To assess a possible role for CD1a in mechanisms of systemic skin disease, we generated a human CD1a transgenic mouse (CD1a-Tg), with the human *CD1A* gene locus inserted by microinjection, akin to the published model^27^ (polymerase chain reaction (PCR) genotyping Supplementary Fig. 1A). We went on to phenotype the mice and determine whether CD1a protein expression followed the expected profile (Fig. 1A). Human dermal DC (dDC) subsets have been reported to express CD1a, and LCs are constitutively CD1a^high^. Transgenic CD1a protein was detected, constituting 4.2% (±1.79) of total ear skin cells and 23.6% (±6.68) of CD45 + cells, expressed by dDCs and LCs (Fig. 1A, B). Cutaneous CD1a protein expression was further characterized by immunofluorescence, revealing characteristic LC epidermal location and dendrites (Fig. 1C). We observed CD1a expression within the thymus predominantly by a proportion of CD4^+^CD8^+^ double positive thymocytes (Supplementary Fig. 1B). CD1a + DCs and LCs were detected in the skin-draining cervical lymph node, while minimal CD1a expression was detected in the blood, spleen, gut or lung (Supplementary Fig. 1C). Additionally, we confirmed transcriptional control of CD1a expression akin to human regulation whereby incubation of CD1a-Tg bone marrow cells and separated bone-marrow-derived CD115 + monocytes with GM-CSF induced CD1a expression. CD1a expression was observed on loosely adherent (enriched for BM-derived macrophages) and floating (enriched for BM-derived DCs) bone-marrow-derived cells (Fig S1D). CD1a-Tg mice showed no aberrant skin inflammation at steady state; however, we did observe a baseline increase in immune populations within the skin, including T cells and LCs (Fig. 1D). In summary, we have generated a CD1a transgenic mouse that displays CD1a expression in a manner phenotypically analogous to human tissue.

### Characterization of CD1a antibodies for neutralization potential

In addition to establishing a CD1a-Tg line to investigate mechanisms of CD1a-dependent inflammation, we screened and characterized a large panel of anti-human CD1a antibodies for neutralization potential. The antibodies were generated by UCB and screened for specific CD1a binding, as detailed in the methods.

**Fig. 2:**
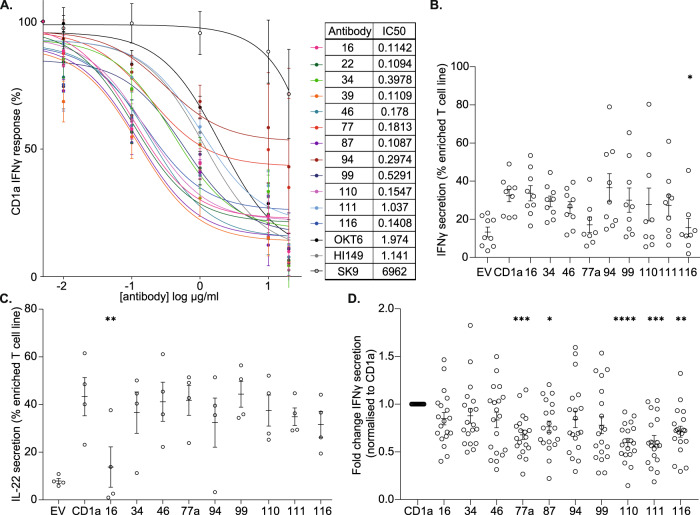
Characterization of anti-CD1a antibodies in the inhibition of T-cell responses. **A** Dose titration curve of polyclonal T-cell IFNγ response with increasing concentration of a refined panel of anti-CD1a antibody (0.01–10 µg/ml) as determined by ELISpot (*n* = 6 donors, mean ± SD). And table of half maximal inhibitory concentration (IC50) values (µg/ml) calculated for the panel of newly generated anti-CD1a antibodies and commercial antibodies (OKT6, HI149 and SK9). *n* = 6 biologically independent blood donors for all groups examined over 4 independent experiments. **B**–**D** Characterization of anti-CD1a antibodies in the inhibition of CD1a-restricted T cells. n represents biologically independent CD1a-restricted T-cell lines. **B**, **C** Cytokine secretion response of CD1a-restricted T cells induced by empty-vector (EV)- or CD1a-transfected (CD1a)-K562 presenting endogenous ligands. Inhibition of IFNγ (**B**) and IL-22 (**C**) was assessed for the panel of anti-CD1a antibodies by flow cytometry. **D** IFNγ secretion response of CD1a-restricted T cells induced by CD1a-coated beads presenting endogenous ligands. Inhibition was assessed for the panel of newly generated anti-CD1a antibodies by flow cytometry. **B** *n* = 9 examined over 4 independent experiments. **C** *n* = 4 examined over 2 independent experiments. **D** *n* = 19 examined over 4 independent experiments. One-way-ANOVA with Dunnett’s or Tukey’s test, *P < 0.05; **P < 0.01; ***P < 0.001; ****P < 0.0001 where asterisk (*) indicates significance on comparison to “CD1a”, mean ± SEM). Exact *p*-values are recorded in Supplementary Table 2. Source Data are provided as a Source Data file.

CD1a binders were evaluated for blocking capacity using a model antigen presentation system. Polyclonal human T cells, derived from the peripheral blood mononuclear cells (PBMC) of healthy donors, were co-cultured overnight with CD1a-transfected K562 cells (CD1a-K562) or control empty-vector K562 (EV-K562)^28^. K562 were incubated with the mouse- and rabbit-derived antibodies, and resulting T-cell activation, secretion of IFNγ, was measured by Enzyme-linked immunosorbent spot (ELISpot) (Supplementary Fig. 2A). Commercially available anti-CD1a blocking, OKT6 and HI149, and non-blocking, SK9, antibodies were included in the assessment (Supplementary Figure 2B). A number of the newly generated anti-CD1a antibodies successfully blocked CD1a-dependent T-cell activation. To further refine the antibody panel, ELISpot was used to perform a dose titration of the top-performing neutralizing anti-CD1a antibodies (Fig. 2A). A number of the newly generated antibodies outperformed blocking antibodies HI149 and OKT6, displaying lower IC50 values (Fig. 2A).

To reduce the inevitable variability associated with polyclonal T-cell assays and aid antibody panel refinement, we generated enriched human CD1a-autoreactive T cells. CD1a-restricted T cells were isolated by fluorescence-activated cell sorting (FACS), using cytokine secretion assays to identify CD1a-responsive T cells based on specific cytokine production (IFNγ or IL-22), and expanded in vitro. The ability of the anti-CD1a antibody panel to block CD1a-dependent secretion of IFNγ and IL-22 was assessed upon co-culture of enriched T cells with EV-K562 or CD1a-K562, using the corresponding secretion assays. Anti-CD1a antibody OX116 was shown to inhibit CD1a-dependent IFNγ production (Fig. 2B); and antibody OX16 was shown to inhibit CD1a-dependent secretion of IL-22 (Fig. 2C). Additionally, CD1a-restricted T cells were co-cultured with CD1a-coated beads as a reductionist model system to minimize any potential confounding target cell effects. Anti-CD1a antibodies OX77a, OX87, OX110, OX111 and OX116 significantly reduced the CD1a-dependent secretion of IFNγ (Fig. 2D).

### CD1a epitope analysis

The above human in vitro analyses identified anti-CD1a antibody clones OX16, OX77a, OX110, OX111 and OX116 as the most efficacious high affinity blockers of CD1a antigen presentation.

**Fig. 3:**
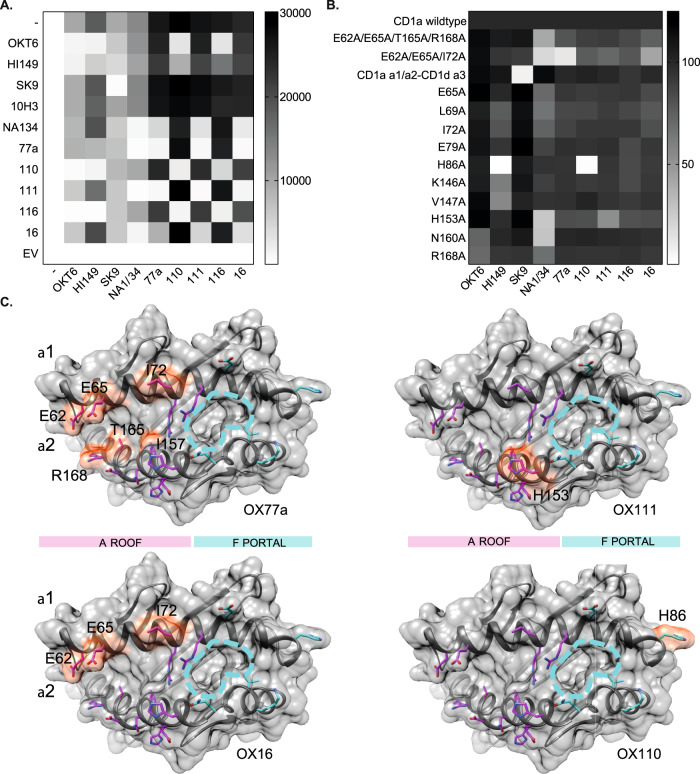
CD1a epitope analysis. **A** Matrix heatmap representation of CD1a antibody binding by flow cytometry as measured by CD1a-AF647 mean fluorescence intensity (MFI). Before staining of CD1a-K652 with anti-CD1a antibodies conjugated to fluorophore AF647 (horizontal axis), the relevant purified antibodies (vertical axis) were incubated with the cells to assess interference in CD1a binding of the AF647-conjugated antibodies. Greyscale shows degree of binding, with the tone in the top row (−) indicating no interference. Representative of 3 independent experiments. **B** Matrix heatmap representation of CD1a antibody binding to CD1a mutant proteins as represented by normalized CD1a-AF647 MFI. Biotinylated CD1a mutant proteins and wild-type CD1a proteins were conjugated to streptavidin magnetic beads and binding of the panel of AF647-conjugated anti-CD1a antibodies was determined by flow cytometry. Greyscale shows degree of binding as a percentage of parent CD1a minor allele. Examined over 4 independent experiments. **C** Summary of CD1a mutants that affect binding of anti-CD1a antibodies OX77a, OX111, OX16, OX110. The top surface of CD1a (grey) is formed by α 1 and α 2 helices. The approximate area of A’ roof/F’ portal is marked with pink and cyan bars. The side chains of residues mutated to alanine are shown in magenta (A’ roof) or cyan (F’ portal). Mutated residues that had a negative impact on binding to the corresponding Ab are labelled and their surface is coloured in red.

To further characterize the top-performing anti-CD1a antibodies, we undertook epitope binding analyses (Fig. 3A-C). CD1a-K562 were incubated with purified anti-CD1a antibodies (*Y*-axis Fig. 3A, 25 µg/ml), the unbound antibody was then washed away, and Alexa-Fluor-647-conjugated forms of the antibodies were then incubated with the cells (*X*-axis Fig. 3A, 10 µg/ml). Mean fluorescent intensity (MFI) was used to assess the degree of conjugated antibody binding. The results indicated that antibodies HI149, OKT6, OX110 and OX116 may have overlapping or closely associated epitopes, and a second group containing antibodies NA1/34, OX77a, OX111 and OX16 may have closely related binding sites.

To further investigate the anti-CD1a antibody epitopes, we generated alanine mutations in CD1a^29^. The panel of tested CD1a variants consisted of single point mutations and multiple mutants, as illustrated in Fig. S3A, and a chimeric variant where the α1 and α2 domains of CD1a were fused to the α3 domain of human CD1d^30^. Biotinylated wild-type (WT) and mutant proteins were bound to streptavidin beads and stained with anti-CD1a antibodies to assess epitope binding. The heatmap depicts the percentage binding normalized to WT CD1a MFI for each antibody tested (Figs. 3B and S3B). Based on the effect of the mutations, we observed two patterns of antibody binding. Antibodies OX77a, OX111 and OX16 appear to target the A’ roof of CD1a, a semi-rigid platform above the lipid-binding groove, which is frequently recognized by autoreactive antigen-independent T cells^29,31^. The epitope of OX110 appears to encompass residues around the F’ portal, the solvent-exposed part of the cleft of CD1a where T cells can potentially contact lipid ligands in an antigen-specific manner. Although none of the mutants tested showed a major impact on the interaction with OX116, the competitive binding assays suggested that both OX110 and OX116 target the region surrounding the F’ portal and might block lipid-specific T cells (Fig. 3C). Additionally, we observed a third anti-CD1a binding mode—SK9 appears to bind on the α3 helix of CD1a as CD1a/d chimeric mutant binding was lost (Figs. 3B and S3A). This is of note, given the non-blocking properties of this antibody.

### Anti-CD1a antibodies prevent imiquimod-induced skin inflammation

Daily topical skin application of imiquimod (5% imiquimod cream, Aldara) is used as a model of cutaneous inflammation. Imiquimod is a TLR7 (and TLR8 in humans) agonist used for the treatment of superficial basal cell carcinomas, actinic keratoses and some viral skin infections. A range of cutaneous adverse effects has been described, including psoriasis, dermatitis, erythema multiforme, vesiculation, alopecia, lichenoid reactions, lupus erythematosus, neutrophilic diseases, oral erosions and Stevens-Johnson syndrome^32–41^, which underlie the murine inflammatory skin model.

**Fig. 4:**
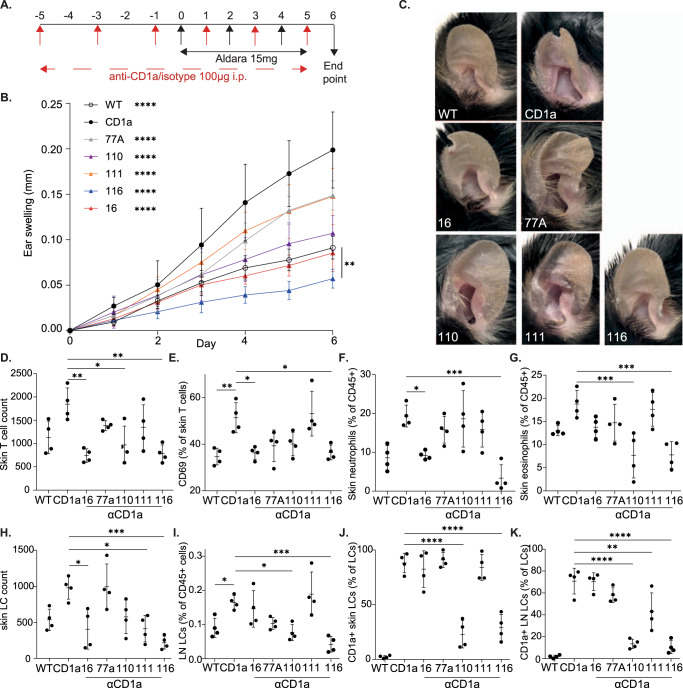
Characterization of anti-CD1a antibodies in vivo. **A** Schematic of imiquimod-induced skin inflammation and anti-CD1a preventative administration. **B** Daily measurement of ear swelling induced by imiquimod treatment of wild-type (WT) and CD1a transgenic mice (CD1a) injected i.p. with mouse IgG1 isotype control and CD1a transgenic injected with the refined panel of anti-CD1a antibodies as in the schematic panel **A**. Mean ± SD is shown. n represents biologically independent animals in each group. **B** *n* = 6 for all groups examined over 3 independent experiments. Two-way-ANOVA with Tukey’s test, **P < 0.01; ****P < 0.0001 indicates significance at day 6 on comparison to “CD1a” or as shown. **C** Representative images of inflammation on day 6 of the imiquimod treatment of wild-type (WT) and CD1a transgenic mice (CD1a) injected i.p. with mouse IgG1 isotype control and CD1a transgenic injected with the refined panel of anti-CD1a antibodies as in the schematic panel **A**. **D**–**G** Flow cytometric analysis of ear skin of mouse IgG1 isotype treated wild-type (WT) and CD1a transgenic (CD1a) and CD1a transgenic injected with the refined panel of anti-CD1a antibodies following the preventative model of administration (panel **A**). Skin T cells were enumerated (/ear) (**D**) and assessed for cell surface CD69 expression (**E**) and skin neutrophil (**F**) and eosinophil (**G**) frequency was determined. Skin (**H**) and draining lymph node (LN) (**I**) LCs were enumerated and assessed for cell surface CD1a expression (**J**–**K**). **D**–**K** *n* = 4 examined over 3 independent experiments. One-way-ANOVA with Tukey’s (**D**–**F**, **H**, **J**, **K**) or Dunnett’s test (**G**, **I**), *P < 0.05; **P < 0.01; ***P < 0.001; ****P < 0.0001. Exact *p*-values are recorded in Supplementary Table 3. Source Data are provided as a Source Data file.

Anti-CD1a antibodies were administered by intraperitoneal injection (i.p.) at 48 h intervals before and during the application of imiquimod to the ear to investigate CD1a-dependence (Fig. 4A). Daily ear thickness measurements revealed the level of inflammation observed in the CD1a-Tg mice was significantly higher than WT counterparts (Fig. 4B), consistent with a previous report^18^. Furthermore, all anti-CD1a antibodies administered reduced ear thickening, and by the experimental endpoint CD1a-Tg mice treated with antibodies, OX16 and OX110 showed a reduction of inflammation to WT level. Strikingly, antibody OX116 reduced the level of CD1a-Tg ear skin inflammation significantly below that of WT skin (Fig. 4B and Fig. S4). Additionally, greater ear thickening, reddening and scaling were observed in the CD1a-Tg mice, which was abrogated in the presence of CD1a blockade (Fig. 4C).

We next sought to analyze the contribution of cutaneous immune populations to imiquimod-induced CD1a-dependent ear inflammation. We found that skin T-cell infiltration was elevated following imiquimod application to the CD1a-Tg compared to WT, and the frequency of this population was reduced by the anti-CD1a antibodies, in particular, antibodies OX116, OX16 and OX110 (Figs. 4D, S4B). Furthermore, activation marker CD69 was increased on skin T cells in the CD1a-Tg in response to imiquimod, and was inhibited by some of the anti-CD1a antibodies, in particular OX116 and OX16 (Fig. 4E). Neutrophils are known to be infiltrating cells for a number of inflammatory disorders, including psoriasis, atopic dermatitis and the murine imiquimod model. Here we found an elevated neutrophil frequency in the skin upon imiquimod treatment and a further increase in the CD1a-Tg, which was significantly reduced with anti-CD1a antibodies OX116 and OX16 (Fig. 4F, S4B). We also noted the reduction in skin eosinophils in response to the antibodies, which is of interest given the known role of eosinophils in atopic dermatitis and, more recently psoriasis (Figs. 4G, S4B). To determine whether the CD1a-dependent imiquimod activation of T cells was due to imiquimod acting as a CD1a ligand, we performed an isoelectric focusing analysis. No difference was detected in the migration of CD1a bands of the mock- and imiquimod-loaded samples, suggesting that imiquimod does not bind directly to CD1a, or has no impact on the CD1a isoelectric point on binding (Supplementary Fig. 5).

Langerhans cells (CD11c^+^ Langerin^+^) were also increased in the skin upon imiquimod challenge of the CD1a-Tg, compared to WT, as has been observed in some human inflammatory skin disorders^42,43^. With the administration of antibodies OX16, OX116, OX111, skin LC count was diminished (Fig. 4H, S4B). OX110 showed a non-significant trend towards skin LC reduction. We analyzed the cervical lymph node (LN) for the presence of CD11c^+^ Langerin^+^ LCs and found an increased number of LCs in the CD1a-Tg LN, compared to WT. However, enhanced migration to the LN did not appear to explain the reduction in skin LCs for mice treated with antibodies OX110 and OX116 (Fig. 4I). As the predominant CD1a-expressing population, we assessed the effect of antibodies on LC CD1a expression, and observed that antibodies OX110 and OX116 reduced staining (Fig. 4J, K). This was explained by in vivo administration of anti-CD1a OX110 and 116 competing with ex vivo flow cytometry detection antibody (clone HI149) as suggested in vitro (Fig. 3A), but not distal clone SK9 (Supplementary Fig. 6). It is of note that the LN-derived LCs expressed less CD1a per cell than those of the skin (Fig. 4J, K).

### Anti-CD1a antibody cytotoxicity

**Fig. 5:**
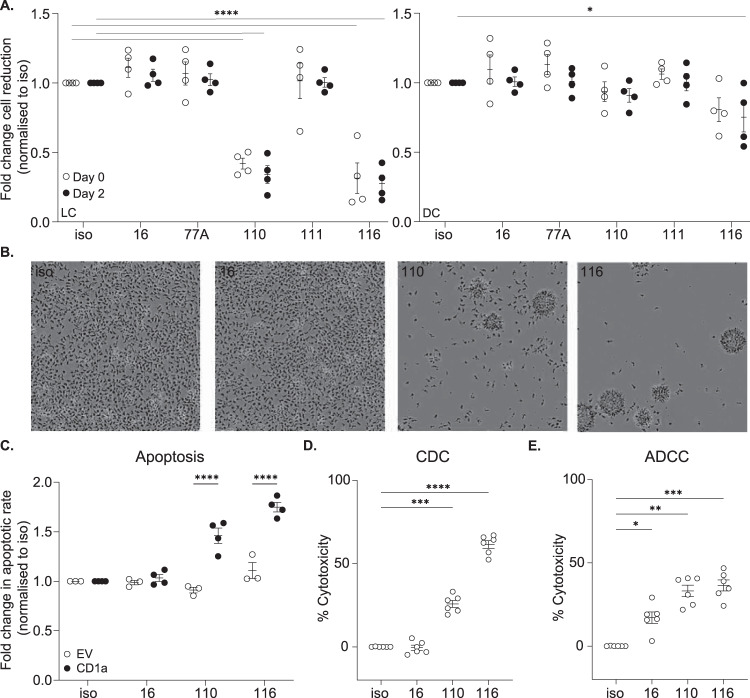
Analysis of anti-CD1a-antibody-induced phenotype change and cytotoxicity. **A** Anti-CD1a antibodies or mouse IgG1 isotype control (iso, 5 µg/ml) were incubated with monocyte-derived Langerhans cells (LC) (left panel) and monocyte-derived dendritic cells (DC) (right panel) as indicated for 5 days with antibodies added on day 0 or day 2 and fold change in cell culture confluency was calculated in relation to the isotype control as measured by percentage confluence using Incucyte live cell imaging. *n* = 4 biologically independent blood monocyte donors examined over 2 independent experiments. Two-way-ANOVA with Dunnett’s multiple comparisons test, *P < 0.05, ****P < 0.0001, mean ± SEM. **B** Representative images of monocyte-derived LCs. data shown representative of 2 independent experiments. **C** K562-CD1a (CD1a) or K562-Empty vector (EV) were incubated with anti-CD1a antibodies for 24 h and stained for Annexin V and analyzed by flow cytometry. *n* = 4 independent cultures of K562 cells, examined over 2 independent experiments. **D** Flow cytometric analysis of complement-dependent cytotoxicity (CDC). K562-CD1a cells were incubated with 10% normal human serum for 3 h at 37 °C in the presence of either 5 µg/ml isotype control antibody or indicated antibodies. Percentage cytotoxicity was calculated in relation to a reference population of untreated K562 and was normalized to isotype control-treated cells. *n* = 6 biologically independent human serum donors examined over 2 independent experiments. **E** Flow cytometric analysis of antibody-dependent cell-mediated cytotoxicity (ADCC). K562-CD1a cells were co-cultured with PBMC at 1:50 ratio for 5 h at 37 °C in the presence of either 5 µg/ml isotype control antibody or indicated antibodies. Percentage cytotoxicity was calculated in relation to a reference population of untreated K562 and was normalized to isotype control-treated cells. n = 6 biologically independent PBMC donors examined over 2 independent experiments. **C**–**E** One-way-ANOVA with Tukey’s test, *P < 0.05; **P < 0.01; ***P < 0.001; ****P < 0.0001 where aterisk (*) indicates significance on comparison to “CD1a”, mean ± SEM). Exact *p*-values are recorded in Supplementary Table 4. Source Data are provided as a Source Data file.

To investigate the cause of apparent LC depletion in vivo (Fig.4H, I), anti-CD1a antibodies were incubated with CD1a-K562 or EV-K562 in vitro for 48 h and the impact on CD1a-expressing cells analyzed. It was demonstrated that OX110 and OX116 reduced CD1a-K562 culture confluency (Supplementary Fig. 7A), with dose dependence (Supplementary Fig. 7B). We went on to assess the impact of the antibodies on primary human CD1a-expressing cells. LC and DC-like cells were generated through 5-day in vitro differentiation of monocytes using cytokines IL-4/GM-CSF/TGF-β and IL-4/GM-CSF, respectively, with the addition of anti-CD1a antibodies on day 0 or 2 of culture. We observed that antibodies OX110 and OX116 reduced the culture confluency of LCs and, to a lesser extent DCs in vitro (Fig. 5 A), and induced an associated clustering morphology (Fig. 5B). To determine the cause of reduced confluency and investigate possible mechanisms of in vivo LC depletion, we employed cytotoxicity assays (Fig. 5C–E). Anti-CD1a antibodies alone were incubated with CD1a-K562, and apoptosis measured by flow cytometry (Fig. 5C); we observed antibody-induced apoptosis of cells incubated with OX110 and OX116. To address further mechanisms that could contribute to LC depletion, we incubated CD1a-K562 with antibodies in the presence of complement-containing human serum (complement-dependent cytotoxicity, CDC, Fig. 5D) or cytotoxic cell-containing PBMC (antibody-dependent cellular cytotoxicity, ADCC, Fig. 5E). Albeit with caveats of use of murine Fc in the presence of human complement and PBMC, it was nevertheless shown that complement (OX110 and OX116) and Fc-mediated cellular cytotoxicity (OX16, OX110, OX116) could induce antibody-mediated cell death of CD1a + cells. Therefore, the depletion of LCs in the skin of CD1a-Tg mice treated with OX110 and OX116, and to a lesser extent OX16, may be explained by direct antibody-dependent killing, complement-mediated and/or cellular cytotoxicity of CD1a^+^ LCs and contribute to the in vivo effect. Via these mechanisms, OX116 could reduce proinflammatory CD1a-dependent and CD1a-independent T-cell responses in the skin and therefore reduce skin inflammation below WT. Depletion of target cells does not explain the reduction of T-cell functional responses shown in Fig. 2, as the CD1a-bead assay (Fig. 2D) would not be affected by depletion. Differential affinity could also not explain the function as the lead antibodies bound with high affinity ((KD OX110 1.97E-09M, OX116 2.89E-09M, OX16 3.92E-10/1.28E-08M (heterogenous)). Therefore, it can be seen that the newly generated OX-antibodies broadly segregate into groups based on functionality and epitope binding regions. Both groups are capable of blocking CD1a-T-cell receptor (TCR) signalling (Supplementary Fig. 2 and Fig. 2), and the group consisting of OX110 and OX116 shows additional phenotypic change and depletion potential in vitro albeit with the caveat of use of murine Fc in the presence of human complement and PBMC (Fig. 5).

### Therapeutic potential of anti-CD1a antibodies

**Fig. 6:**
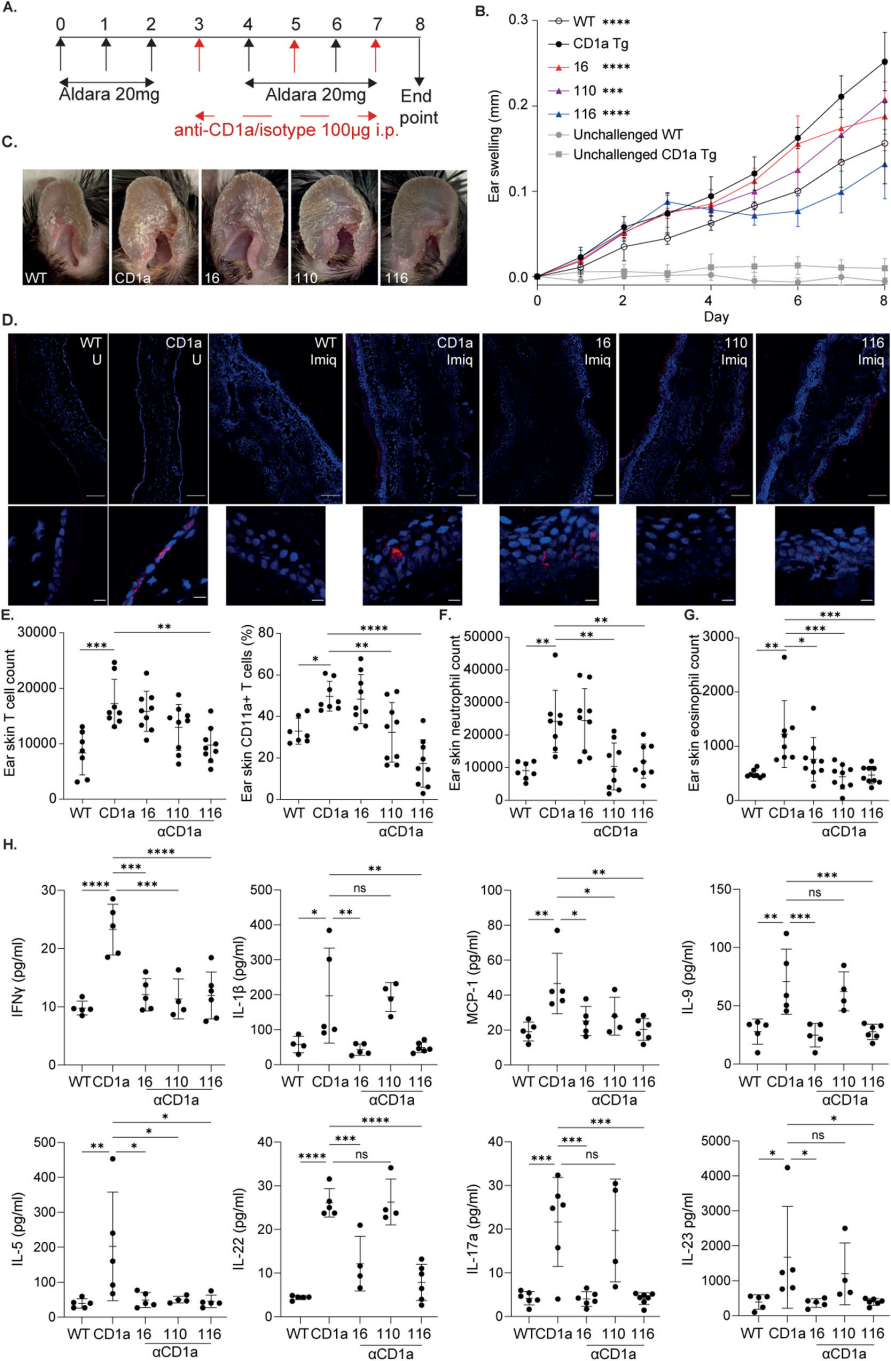
Application of anti-CD1a antibodies in the treatment of imiquimod-induced inflammation. **A** Schematic of imiquimod-induced inflammation with therapeutic anti-CD1a administration. **B** Daily measurement of ear swelling. *n* = 4 (WT), *n* = 6 (CD1a), *n* = 8 (16), *n* = 8 (110), *n* = 10 (116), *n* = 2 (WT unchallenged), *n* = 6 (CD1a unchallenged), examined over 3 independent experiments. Two-way-ANOVA with Dunnett’s test, ***P < 0.001; ****P < 0.0001 indicates significance at day 6 on comparison to “CD1a”. **C** Representative images of inflammation (day 8) induced by imiquimod treatment of wild-type (WT) and CD1a transgenic mice (CD1a Tg) followed by the treatment i.p. with mouse IgG1 isotype control or CD1a transgenic injected with the refined panel of anti-CD1a antibodies as in the schematic panel A (at day 3 arrowpoint). **D** Whole ear (upper panels) and epidermal (lower panels) thickness and CD1a protein expression within ear skin of wild-type (WT) and CD1a transgenic (CD1a) mice treated with imiquimod (Imiq) or untreated (U) visualized by immunofluorescence. Cryosections were stained with DAPI (blue) and anti-CD1a AF-594 (OKT6, red), scale bars 10 µm upper panels and 100 µm lower panels. Data shown representative of 2 independent experiments. **E**–**G** Flow cytometric analysis of ear skin of mouse IgG1 isotype treated wild-type (WT) and CD1a transgenic (CD1a) and CD1a transgenic injected with the refined panel of anti-CD1a antibodies following the treatment model of administration. Skin T cells were enumerated (/ear) and assessed for cell surface CD11a expression (**E**) and neutrophil (**F**) and eosinophil (**G**) enumerated (/ear). **E**–**G** *n* = 7 (WT), *n* = 8 (CD1a), *n* = 9 (16, 110, 116), examined over 3 independent experiments. One-way-ANOVA with Dunnett’s test, *P < 0.05; **P < 0.01; ***P < 0.001; ****P < 0.0001. **H** Skin cytokine levels of mouse IgG1 isotype treated wild-type (WT) and CD1a transgenic (CD1a); and CD1a transgenic injected with anti-CD1a antibodies following the treatment model of administration as measured by cytometric bead array. *n* = 5 (WT, CD1a, 16), *n* = 4 (110), *n* = 6 (116), examined over 3 independent experiments. One-way-ANOVA with Dunnett’s test, *P < 0.05; **P < 0.01; ***P < 0.001; ****P < 0.0001. Mean ± SD is shown. n represents biologically independent animals in each group. Exact *p*-values are recorded in Supplementary Table 5. Source Data are provided as a Source Data file.

To further evaluate the newly generated anti-CD1a antibodies, we tested the three most effective (OX16, OX110 and OX116) in an imiquimod treatment model, where antibodies were introduced after the establishment of inflammation (Fig. 6A). All three antibodies improved clinically-relevant responses rapidly after initiation despite ongoing imiquimod application (Fig. 6B, C and Supplementary Fig. 8). The responses were most marked for OX116, which appeared to reduce ear thickness to below WT (Fig. 6B and Supplementary Fig. 8). Whole skin and epidermal thickening visualized by confocal microscopy (Fig. 6D) confirmed micrometre assessment (Fig. 6B). CD1a protein expression was assessed in the CD1a-Tg epidermis and noted to be reduced, likely through cell death and epitope competition, in OX110- and OX116-treated skin (Fig. 6D). Upon analysis of the cutaneous cellular immune response following the imiquimod treatment model we observed reduced skin T-cell count and activation, and reduced skin neutrophils and eosinophils after the introduction of the antibodies, confirming CD1a involvement (Fig. 6E–G). To investigate the CD1a-dependent mechanisms underlying imiquimod-induced skin inflammation, skin cytokine secretion was assessed by flow cytometry-based multiplex bead assays. CD1a-dependent increases in a broad range of T cell and innate-derived cytokines were detected, including IL-22, IL-17A and IL-23, which have previously been shown to be key regulators of the murine imiquimod model (Fig. 6H and Fig S9). We confirmed the enhancement of T-cell-derived IL-17A in the CD1a-Tg by intracellular flow cytometry staining (Supplementary Fig. 10). The effects of the antibodies OX16 and OX116 on large populations of IL-17A-producing cells would be compatible with effects beyond the CD1a-restricted T-cell subset and argues that the antibodies impact a cascade of inflammation with potential systemic impact, which we address next.

### CD1a is involved in the systemic immune reaction to imiquimod

**Fig. 7:**
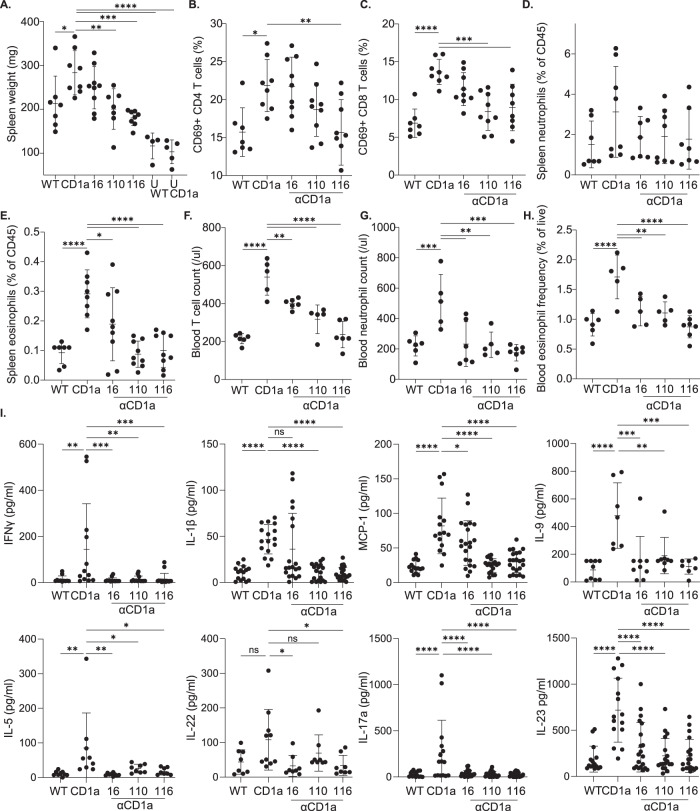
Investigation of the CD1a dependency of the systemic effects of imiquimod application. **A** Spleen weight (mg) measurements on day 8 by imiquimod treatment of wild-type (WT) and CD1a transgenic mice (CD1a) followed by treatment i.p. with mouse IgG1 isotype control or CD1a transgenic injected with the refined panel of anti-CD1a antibodies as in the schematic (Fig. 6A). **B**–**E** Flow cytometric analysis of spleen of mouse IgG1 isotype treated wild-type (WT) and CD1a transgenic (CD1a); and CD1a transgenic injected with the refined panel of anti-CD1a antibodies following the treatment model of administration. Splenic CD4 (**B**) and CD8 (**C**) T-cell CD69 expression was assessed and neutrophils (**D**) and eosinophils (**E**) were enumerated. **F**–**H** Blood cellular analysis of the blood of mouse IgG1 isotype treated wild-type (WT) and CD1a transgenic (CD1a); and CD1a transgenic injected with the refined panel of anti-CD1a antibodies following the treatment model of administration. Circulating T cells (**F**), neutrophils (**G**) and eosinophils (**H**) were enumerated. **I** Plasma cytokine levels of the blood of mouse IgG1 isotype treated wild-type (WT) and CD1a transgenic (CD1a); and CD1a transgenic injected with anti-CD1a antibodies following the treatment model of administration as measured by cytometric bead array. Mean ± SD is shown. n represents biologically independent animals in each group. One-way-ANOVA with Dunnett’s test, *P < 0.05; **P < 0.01; ***P < 0.001; ****P < 0.0001, examined over 3 (**A**–**H** I. IL-9, IL-5, IL-22) or 4 (**I** IFNγ, IL-1β, MCP-1, IL-17a, IL-23) independent experiments. Exact *p*-values and group sizes are recorded in Supplementary Table 6. Source Data are provided as a Source Data file.

The inflammatory symptoms of psoriasis and of imiquimod clinical use extend beyond the skin with flu-like symptoms and spleen enlargement observed in patients with psoriasis and during murine application^44^. The establishment of the cutaneous inflammation model and characterization of efficacious therapeutic antibodies facilitated the evaluation of the contribution of CD1a to this pathway. Strikingly, spleen weight was increased in the imiquimod-treated CD1a-Tg compared to WT, and the antibodies reduced spleen size and weight, consistent with systemic CD1a-dependent effects (Figs. 7A and S11A). Furthermore, the antibodies reduced CD4 and CD8 T-cell activation as determined by CD69 expression (OX116 and OX110, Fig. 7B, C), and splenic neutrophil (non-significant trend) and eosinophil frequencies (OX16, OX110, OX116) (Figs. 7D and E, respectively). We went on to ascertain the impact on circulating immune cells. Similar to the spleen, blood CD4 and CD8 T-cell counts, neutrophilia and eosinophilia were detected in the imiquimod-treated CD1a-Tg mice. This increase was significantly blocked following treatment with anti-CD1a antibodies (Fig. 7F–H and S11B–C). Plasma cytokine levels were assessed on day 8. We observed significant increases in a number of proinflammatory cytokines, including IL-17A, IL-23, IL-1β, MCP-1, IL-9, GM-CSF and IL-5 in the imiquimod-treated CD1a-Tg, which were reduced in the anti-CD1a-antibody-treated groups (Fig. 7I and Supplementary Fig. 11D). Interestingly regulatory cytokines IL-27 and non-significantly IL-10, appeared to decrease in the CD1a-Tg and increase in the antibody-treated groups (Supplementary Fig. 11D).

### CD1a contributes to enhanced inflammation in a dermatitis model

**Fig. 8:**
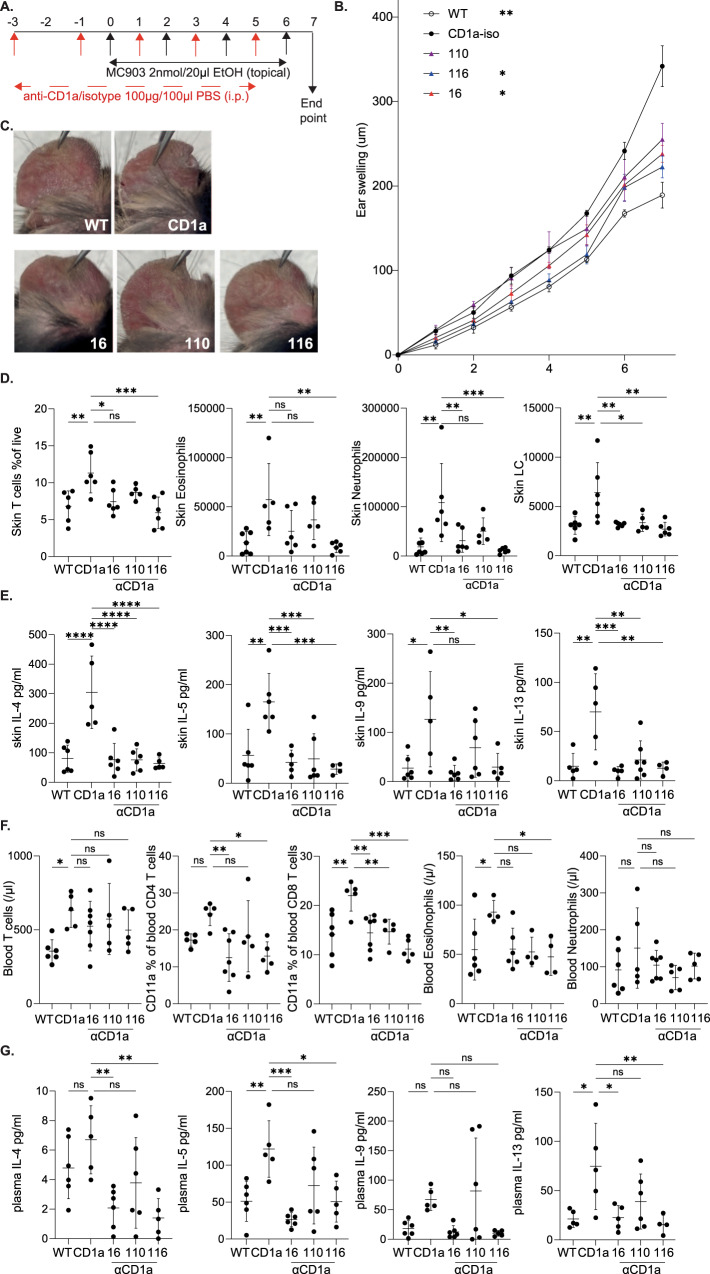
Analysis of the contribution of CD1a to MC903-induced inflammation. **A** Schematic of MC903-induced inflammation with therapeutic anti-CD1a administration. **B** Daily measurement of ear swelling. *n* = 5 for all groups, two-way-ANOVA with Dunnett’s test, **P < 0.01; ****P < 0.0001 indicates significance on comparison to “CD1a” at day 7, examined over 2 independent experiments. **C** Representative images of inflammation (day 7), induced by MC903 treatment of wild-type (WT) and CD1a transgenic mice (CD1a) and countered by the treatment i.p. with mouse IgG1 isotype control or CD1a transgenic injected with the refined panel of anti-CD1a antibodies as in the schematic panel. **D** Flow cytometric analysis of ear skin of mouse IgG1 isotype treated wild-type (WT) and CD1a transgenic (CD1a) and CD1a transgenic injected with the refined panel of anti-CD1a antibodies. Skin T cells, neutrophils, eosinophils and LCs were enumerated (/ear). **E** Skin cytokine levels of mouse IgG1 isotype treated wild-type (WT) and CD1a transgenic (CD1a); and CD1a transgenic injected with anti-CD1a antibodies following the treatment model of administration. **F** Flow cytometric analysis of the blood of mouse IgG1 isotype treated wild-type (WT) and CD1a transgenic (CD1a) and CD1a transgenic injected with the refined panel of anti-CD1a antibodies. Skin T cells were enumerated and assessed for activation marker expression and blood neutrophil and, eosinophils were enumerated. **G** Plasma cytokine levels of mouse IgG1 isotype treated wild-type (WT) and CD1a transgenic (CD1a); and CD1a transgenic injected with anti-CD1a antibodies following the treatment model of administration as measured by cytometric bead array. **D**–**G** *n* = 7 (WT), *n* = 6 (CD1a, 16, 116), *n* = 5 (110), examined over 2 independent experiments. One-way-ANOVA with Dunnett’s test, *P < 0.05; **P < 0.01; ***P < 0.001; ****P < 0.0001. Mean ± SD is shown. n represents biologically independent animals in each group. Exact *p*-values are recorded in Supplementary Table 7. Source Data are provided as a Source Data file.

To evaluate the role of CD1a in an alternative model of skin inflammation, we employed the MC903 experimental dermatitis model. MC903 is a low-calcemic vitamin D3 analogue that is an approach used to treat patients with psoriasis, but can induce dermatitis as a side-effect, and application to murine skin induces dermatitis-like inflammation characterized by spongiosis, skin thickening, hypervascularisation, epidermal hyperplasia, pruritus, immune cell infiltration and dependence on type-2 cytokine pathways including type-2 inducer cytokines TSLP and IL-33^45,46^. MC903 was applied to WT or CD1a-Tg mouse ear skin daily for 7 days (Fig. 8A), and anti-CD1a or isotype control antibody administered intraperitoneally to examine the contribution of CD1a to inflammation. MC903-induced ear skin thickening and inflammation were significantly increased in the CD1a-Tg and reduced by anti-CD1a antibody treatment (Fig. 8B and Supplementary Fig. 12A, B). Skin T cell, eosinophil and neutrophil and LC infiltration was increased in the CD1a-Tg and reduced by administration of anti-CD1a antibodies, in particular OX16 and OX116 (Fig. 8C). Upon skin cytokine analysis, CD1a-dependent type-2 cytokine production was observed (Fig. 8D) in addition to further proinflammatory cytokines (Supplementary Figure 13) and chemokines (Supplementary Fig. 14), which may contribute to the observed immune infiltration. Increased activation of CD4 and CD8 T cells in the draining LN was observed (Supplementary Fig. 12E).

We next sought to determine whether our observation of CD1a-dependent systemic inflammation in the imiquimod model of murine skin inflammation would extend to alternative pathways of inflammation. MC903 induced increased circulating T-cell infiltration and activation that was amplified in the CD1a-Tg (Fig. 8F). Additionally, circulating eosinophil and neutrophil numbers were increased, showing some CD1a dependency (Fig. 8F). Similar trends were observed in the spleen of the MC903 treated mice (Supplementary Fig. 12F–H). To evaluate the CD1a-dependent pathways of systemic inflammation, plasma effector cytokine levels were assessed, and amplification of type-2 cytokine (Fig. 8G) and other proinflammatory chemokine and cytokine production was detected (Supplementary Fig. 14 and Supplementary Fig. 15). Therefore, overall, we suggest a previously unrecognized pathway by which systemic inflammatory immune responses are primed or orchestrated by CD1a in the skin.

## Discussion

A number of skin diseases are associated with co-morbidities and harbour systemic immune abnormalities. Chronic subclinical systemic inflammation is thought to underlie splenomegaly observed in severe psoriasis patients and the development of co-morbidities^47^. Thorough correlative analyses of human sera from individuals with AD, psoriasis and healthy comparators have identified systemic biomarkers of disease. However, mechanisms underlying the connection between cutaneous inflammation and increases in serum cytokines and immune cell infiltration are lacking. Here we propose a previously unappreciated mechanism by which skin inflammation can affect systemic immunity through CD1a-dependent effects, defining the pathway as a potentially broad therapeutic target. Through the generation of a CD1a transgenic mouse and autoreactive human CD1a-restricted T-cell lines, and the characterization of anti-CD1a antibodies, the data presented here demonstrate that CD1a amplifies the cutaneous and systemic response in murine models of skin inflammation. The newly generated anti-CD1a antibodies had clinically-relevant and immunological effects, whether they were blocking or blocking/depleting, suggesting that CD1a lipid presentation to T cells is of importance to skin and systemic inflammation with implications for co-morbidities.

Increased circulating T cells have been reported in the serum of patients with psoriasis and atopic dermatitis^48,49^. Imiquimod-induced skin inflammation resulted in increased T-cell number and activation in CD1a-Tg blood and spleen, in addition to the skin and draining LN, compared to WT. We confirmed CD1a-dependence showing that administration of anti-CD1a not only ameliorates cutaneous inflammation but also systemic inflammation as measured by splenomegaly, cellular infiltration and plasma cytokine secretion. Surprisingly, it has been reported that imiquimod-induced splenomegaly associated with decreased T- and B-cell frequency, whereas expansion of innate cell populations (NK, DC, granulocytes, macrophages) may underlie the increased splenic mass^50^. In addition to effects on the T-cell response to the imiquimod-containing drug Aldara, increased neutrophil and eosinophil responses in the skin, draining LN, spleen and blood were observed in the CD1a-Tg.

Imiquimod-dependent eosinophil infiltration of the skin, LNs and spleen were enhanced in the CD1a-Tg mouse and reduced by the administration of anti-CD1a (OX16, OX110 and OX116). We detected skin and plasma cytokines and chemokines consistent with enhanced eosinophil recruitment in the CD1a-Tg, including IL-5, IL-1β, IL-4, GM-CSF, TNFα, MIP-1α, MIP-1β and eotaxin^51,52^. TLR7 + eosinophils have been shown to release cytotoxic granules and IL-8 upon imiquimod application and thereby activate neutrophils^53^. Neutrophil depletion has been shown to ameliorate the severity of imiquimod-induced inflammation^54^. It is of note that imiquimod application upregulates keratinocyte-derived CXCR2 ligands in the skin, which induce neutrophil production of LTB_4_^54^. LTB_4_ is a lipid mediator and bio-active metabolite of arachidonic acid, which is itself a product of the action of the enzyme phospholipase-A2. We have shown previously that phospholipase-A2 activity generates endogenous neolipid CD1a ligands^13,15–17,31^. Modifications to lipid synthesis pathways may alter CD1a antigen presentation and activate CD1a-restricted T cells, driving subsequent inflammatory sequelae. This may be a route by which imiquimod and similar drugs/disease states induce CD1a-dependent inflammation.

Proinflammatory cytokine production is thought to contribute to the systemic features of skin disease, and there are significant overlapping mechanisms between skin disease and extra-cutaneous inflammatory co-morbidities. However, there are key differences that require further research for intervention; for example, anti-IL-17A is a highly effective psoriasis therapy but has been reported to have no impact on systemic CVD markers in psoriasis patients^55^. We observed significant CD1a-dependent increases in proinflammatory cytokines in CD1a-Tg skin and sera, which overlap with the inflammatory serum profiles of AD and psoriasis patients. We detected significant increases in a broad range of cytokines, both T-cell-derived, for example, T helper (Th)1 (e.g. IFNγ, IL-1β, TNFα), Th2 (e.g. IL-5, IL-19 IL-13), Th3 cytokines (e.g. IL-22, IL-17A, IL-17F), and cytokines from innate/non-hematopoietic sources, including IL-23, type-1 interferon and MCP-1. The cytokines that were increased in the CD1a-Tg and inhibited by anti-CD1a antibodies, have been shown to contribute to the orchestration of a network of proinflammatory effectors and may underlie the amplification seen in the CD1a-Tg skin models. For example, IL-23 acts upstream of IL-17A and is an important driver of skin inflammation in the imiquimod model. Individuals with psoriasis were shown to display elevated IL-17A, and the imiquimod model of skin inflammation is reliant on the induction of IL-17A, which has been reported to act on keratinocytes and induce neutrophil attraction and contribute to skin inflammation^56^. Additionally, imiquimod-induced splenomegaly is significantly reduced by IFNAR1 and TNFR1 deficiency^57^, highlighting the importance of cytokines in the systemic effects of skin disease models.

The effects we observed were inhibited by the administration of anti-CD1a antibodies OX16, OX110 and OX116, suggesting anti-CD1a antibodies can modulate this cutaneous and systemic response. This implicates a CD1a-dependent immune cascade that is wider reaching than initially anticipated. Cytokines produced in a CD1a-dependent manner in the skin may enter the circulation along with the migration of CD1a-expressing and responsive cells during inflammatory events contributing to and inducing blood cytokine levels from CD1a-restricted, T cells, bystander T cells and innate sources. Additionally, although we detected no splenic or blood CD1a expression in these models, given our finding that GM-CSF could induce CD1a expression by bone-marrow-derived cells in vitro, it could be that elevated skin and plasma GM-CSF induced CD1a expression could be further relevant in a more chronic model of inflammation or human disease. Indeed, during severe inflammation such as COPD and IBD, extra-cutaneous expression of CD1a-expressing populations has been identified in the lung and gut^25,58^.

In C57BL/6 mice, imiquimod-induced inflammation is dependent on the IL-23-IL-17 axis involving Th17 cells, gamma-delta T cells, neutrophils, mononuclear phagocytes and ILC3, and can be controlled by Tregs through restraint of an IFN-I-driven CD8 + T-cell response akin to human psoriasis^44,59^. Additionally, topical application of imiquimod induces migration of LCs from the treated skin into the draining lymph nodes^60^. TLR7-agonist imiquimod also has TLR-independent effects, thought to be mediated by adenosine receptor modulation augmenting inflammation and acting synergistically with the TLR-dependent mode of action^61^. MC903 application to the skin recapitulates important hallmarks of atopic dermatitis inflammation typified by pruritus, erythema, skin thickening, fine scaling, hypervascularization, spongiosis, strong immune cell infiltration and epidermal hyperplasia and type-2 cytokine production^45^. The MC903-dependent WT dermal inflammatory infiltrate consists of eosinophils and CD4^+^ T cells, DCs, ILC2, granulocytes and mast cells. Skin treated with MC903 shows increased levels of TSLP, type-2 cytokines and further proinflammatory cytokines e.g. IL-8 and IFNγ^45^. Notably, LCs have been shown to contribute to MC903-induced AD inflammation^62^ and undergo hyper-proliferation in AD skin^63^, which would be consistent with the CD1a-dependent enhancement that we observed.

Although systemic changes were observed in response to imiquimod, there were no distal skin manifestations. This may indicate that imiquimod-induced self-lipid ligands or an otherwise altered local inflammatory milieu favouring functional activity of recruited T cells. This could include alterations in local cytokine production, changes in permissive/non-permissive lipid ligands or changes in regulatory T-cell frequency or function^31,59,64^ and immunomodulatory cytokines, as suggested by our observations of reduced serum IL-10 and IL-27 in the CD1a-Tg. However, altered basal cutaneous immune populations were observed in the CD1a-Tg, including small but significant increases in T cells, eosinophils, neutrophils and Langerhans cells at steady state in comparison to WT littermates. This may represent a CD1a-dependent poised setting for surveillance in the skin and may contribute to the rapid amplification of inflammation in the CD1a-Tg upon challenge.

The results reported here and previously published by our laboratory and others lead us to propose that CD1a contributes to the initiation and amplification of local and systemic events during skin inflammation. Activated neutrophils and keratinocytes produce phospholipase-A2 (PLA2), which may result in endogenous neolipid CD1a antigen generation. There are likely to be additional sources of CD1a ligands in the ensuing cutaneous damage of the inflammatory response, where spatial regulation of CD1a engagement is disrupted. In addition to endogenous antigens, it is likely exogenous lipid antigens may contribute to different forms of human skin disease. In contact dermatitis, urushiol^18^ and fragrances^19^ have been defined, and dideoxymycobactin has been identified as a CD1a ligand with relevance to mycobacterial infections^65,66^. Furthermore, dysregulated lipid metabolism and altered lipid profiles have been detected in AD^67^ and psoriasis^68^, which may provide a source of stress-induced CD1a ligands and displacement of natural blockers^64^. APC-activated CD1a-restricted T cells produce a wide range of effector cytokines to orchestrate skin inflammation, including cytokines that induce further eosinophil and neutrophil infiltration, monocyte differentiation and amplify bystander and CD1a-restricted and MHC-restricted T-cell activation and differentiation. Upon imiquimod binding, TLR7 + LCs produce IL-23 and IL-15^69^, and TLR7 + CD1a + DCs produce IFNα and IL-23^11^, which support T-cell activation and inflammatory cytokine production. Egress of immune cells, including CD1a-restricted T cells and TLR7 + CD1a + DCs, into the circulation along with the release of CD1a-induced cytokines, may contribute to systemic inflammation and co-morbidities associated with skin disease. Under a chronically activated state, increases in certain proinflammatory cytokines could induce extra-cutaneous CD1a expression and further contribute to CD1a-dependent pathways of systemic inflammation and inflammatory skin disease co-morbidities.

We observed that CD1a-antibody-dependent depletion of LCs was associated with reduced skin inflammation upon administration of antibodies OX110 and OX116, which may be of therapeutic importance to the treatment of inflammatory conditions. In vitro binding of OX-anti-CD1a antibodies was shown to variably induce direct apoptosis, associating with a clustering morphology (OX110, OX116), complement-dependent cytotoxicity (OX110, OX116) and antibody-dependent cellular cytotoxicity (OX16, OX110, OX116). Such mechanisms may underlie the reduction in LCs seen in vivo in the skin (OX16, OX110, OX116) and draining lymph nodes (OX110, OX116) treated with anti-CD1a antibodies. The depletion effects in all assays performed and in vivo were greatest following treatment with OX116, which may contribute to the profound reduction in skin inflammation by OX116. However, depletion does not fully explain the effects seen; indeed, OX16 and OX116 had comparable effects on cytokine production, and bead-based assays were independent of APC depletion, suggesting a key role for CD1a-TCR signal neutralization. The use of different Fc regions may modulate such effector activity—these will be important to test, alongside Fab fragments. Bechan et al. previously showed the potential of anti-CD1a antibodies in depleting CD1a-expressing cells^70^ as such this is a key consideration for therapeutic implementation.

Our epitope analysis highlights the potential therapeutic importance of epitope binding sites; the anti-CD1a antibodies fell into two groups based on binding site and resultant effector function. The epitope site may facilitate a crosslinking/agglutination-like cell death seen with antibodies OX110 and OX116 but not OX77a, OX111 and OX16, which were primarily blocking antibodies lacking direct antibody-induced apoptosis. This may explain the increased killing of CD1a-transfected K562 and monocyte-derived LCs, as both cell types express high levels of CD1a, compared to monocyte-derived DCs. However, it will be important to investigate the role of bivalency and the Fc region in such an effector function. Thus, preferential depletion of CD1a-high LCs may leave dDC intact, preserving antigen presentation in the skin. Additionally, we observed that different anti-CD1a binding antibodies had a differential impact on cytokine blockade. We observed that OX16 was highly effective at blocking IL-22 production, where for example, OX116 was a more efficacious blocker of IFNγ production. There could be a number of explanations for this observation, including specific lipid ligands and modes of TCR blockade that define the cytokine produced and the roles of the Fc region, which will require further investigation.

Human CD1a-deficiency has been identified and linked to increased susceptibility to tuberculosis^71–73^. However, abnormalities in skin barrier function and other infections have not been reported, pointing to the therapeutic targeting of CD1a. Analysis of serum of individuals with CD1a-deficiency is yet to be performed, and our mouse models provide the possibility to assess systemic CD1a-dependent immunity during inflammation. In summary, we propose a mechanism by which CD1a, a primarily cutaneous antigen presentation molecule, drives systemic inflammation following a challenge to the skin, which may contribute to co-morbidities associated with skin diseases. We report that targeting CD1a with neutralizing antibodies has therapeutic potential for skin and systemic disease.

## Methods

### Mice

Mouse experiments were approved by the local Ethical Review Committee at the University of Oxford, and performed under license from the UK home office (project license numbers P0A3EF7BE, PP4306634, PBA43A2E4) in accordance with the Animals (Scientific Procedures) Act, 1986. Mice were bred and maintained in a specific pathogen-free facility in individually ventilated cages in an ambient temperature- and humidity-controlled room with a 12 h light/12 h dark cycle under standard housing conditions with continuous access to food and water. In individual experiments, mice were matched for age, sex and background strain with wild-type litter mates used as matched controls. Experimental/control animals were co-housed during breeding but kept separate during experimental work to avoid grooming effects.

### *CD1a* transgenic mouse generation

Mice were generated by the Wellcome Trust Centre for Human Genetics, Oxford. A 5.7 kb genomic fragment encompassing the entire *CD1A* gene, including 0.8 kb of upstream sequence and 0.8 kb of downstream sequence, was amplified from human genomic DNA by PCR using primers 5’-ATGGTACCAAGAGGAATGTAAATGTGTCCGGC-3’ and 5’-AAGCGGCCGCGATCATGTTAACCAAGGTCAGGAA-3’ and subcloned into the Litmus28 vector (NEB) via the KpnI and NotI sites incorporated into these PCR primers. After sequence verification of the coding exons, the fragment transgene was excised from the vector backbone, purified and resuspended at 2 ng/ul in microinjection buffer (10 mM Tris-HCl, pH 7.4, 0.25 mM EDTA) and microinjected into a pronucleus of fertilized zygotes prepared from C57BL/6 J mice. After overnight culture, the resulting 2-cell embryos were surgically implanted into the oviduct of pseudopregnant CD1 foster mother and carried to term. Transgenic offspring were identified by PCR using transgene-specific primers and bred as individual lines with wild-type C57BL/6 J mice.

### CD1a genotyping

Crude genomic DNA preparation was performed on ear notch samples from CD1a transgenic mice. One hundred microlitres of DirectPCR ear lysis buffer (Viagen) supplemented with 0.4 mg/ml proteinase K (Sigma) was added to ear notches and incubated at 55 °C overnight. Enzymes were then heat-inactivated at 85 °C for 1 h. The samples were centrifuged to pellet debris, and the lysate was transferred to a clean tube. One microlitre of lysate was used as a template for genotyping. The PCR reaction used for genotyping is detailed in Supplementary Tables 14 and Supplementary Table 15. PCR products were loaded onto a 1% TAE agarose gel with SyberSafe, electrophoresis run and the gel imaged under UV. If the expected band at 655 bp was detected, mice were considered positive for the *CD1A* transgene.

### Generation of therapeutic anti-CD1a antibodies: UCB antibody discovery

Female Balb/C mice (028 Balb/c from Charles River) 11–13 weeks old were immunized with syngeneic cells transfected with human CD1a and mouse β2M and Female New Zealand White rabbits (model code 444, strain HsdIf:NZW from Envigo) 12–16 weeks old were immunized with syngeneic cells transfected with human CD1a and rabbit β2M. Mice were housed in conventional cages with temperatures maintained between 19 and 23 °C and a humidity of around 55 ± 10%. Rabbits were housed in floor pens with temperatures in the room maintained between 16 and 20 °C with the same humidity. For both mice and rabbits, 12 h light 12 h dark are cycled on the system from 7 to 7.

Sera were monitored for binding to human CD1a before the mice and rabbits were euthanised by schedule 1 cervical dislocation and intravenous overdose of anaesthetic, respectively, and PBMC, spleen, bone marrow and lymph nodes harvested. Supernatants from memory B-cell cultures were screened for their ability to selectively bind human CD1a. Antibody binding enzyme-linked immunosorbent assay (ELISA) was performed with an extracellular domain protein preparation (AA 1-300) and FACS binding analysis used CD1a-transfected Expi293 cells (Thermo Fisher Scientific) and C1R cells and endogenous CD1a-expressing MOLT4 cells. Furthermore, non-specific CD1 binding was excluded by assessment of antibody binding to C1R cells expressing CD1b, CD1c or CD1d. Wells demonstrating binding in the above assays were progressed for V-region recovery. Single antigen-specific B cells were isolated via the fluorescent foci method using protein as the target antigen^74^. The fluorescent foci system was also used to directly isolate CD1a-specific plasma cells from bone marrow. Following reverse transcription (RT) and PCR of the picked B cells^75^, antibody V-region genes were cloned into separate heavy and light chain expression vectors, and recombinant rabbit and mouse IgG generated using a HEK-293 expression system^75^. In total, 119 V-regions were cloned and registered. Recombinant cloned antibodies were then further characterized by ELISA, flow cytometry and SPR. Antibodies demonstrating binding in the above assays and <100 nM affinity were selected for purification. Cell culture supernatants were purified using Protein A affinity purification. Purified samples were buffer exchanged in to 10 mM PBS pH 7.4 and analyzed for its recovery and purity using UV spectroscopy, analytical size exclusion chromatography, SDS Page electrophoresis and LAL endotoxin assay, respectively.

DNA encoding the heavy and light chain V-regions of 77 A, 110, 111 and 116 on a mouse IgG1 backbone was synthesized at ATUM, expanded and expressed in a CHO transient expression system in-house. The antibodies then underwent Protein A purification and preparative size exclusion and were tested in in vivo assays.

### Cell lines

Empty-vector-transfected K562 (EV-K562) and CD1a-transfected K562 (CD1a-K562) cells (a gift from B. Moody, Brigham and Women’s Hospital, Harvard Medical School, Boston, MA^22^) were maintained in RPMI 1640 medium supplemented with 10% FCS, 100 IU/ml penicillin, 100 μg/ml streptomycin (Sigma–Aldrich), 2 mM L-glutamine (Gibco), 1× nonessential amino acids (NEAAs) (Gibco), 1 mM sodium pyruvate (Gibco), 10 mM HEPES (Gibco), 500 μM 2-mercaptoethanol (Gibco) and 80 μg/ml G418 antibiotic (Thermo Fisher Scientific). Expi-293 cells used in the characterization/validation of CD1a antibodies were sourced from Thermo Fisher Scientific.

### ELISpot

ELISpot assay (IFNγ ELISpot kit, Mabtech, AB) was used to detect activation-induced cytokine secretion from polyclonal T cells upon co-culture with model CD1a-expressing antigen-presenting cells. PBMCs from healthy donor blood were isolated by density gradient (Lymphoprep) and T cells purified using anti-CD3 magnetic bead sorting following the manufacturer’s protocol (MACS, Miltenyi). All study participants gave fully informed written consent. PBMCs were isolated from healthy adult donors under local ethics approval (National Research Ethics Service Committee South Central, Oxford C, 14/SC/0106). T cells were then cultured for 3 days with IL-2 (200U/ml) to expand in number prior to overnight co-culture with unpulsed/endogenous lipid-bound CD1a-transfected K562 (CD1a-K562) or control empty-vector-transfected K562 (EV-K562) at a ratio of 2.5 × 10^4^ K562 to 5 × 10^4^ polyclonal T cells. To assess the functionality of the anti-CD1a antibodies, K562 were incubated with 10 µg/ml anti-CD1a antibodies 1 h prior to and during co-culture with polyclonal T cells in an anti-IFNγ capture antibody coated ELISpot plate (Millipore Corp., MA). IFNγ secretion was detected with a biotinylated anti-IFNγ detection antibody and visualized with streptavidin-alkaline phosphatase development. The resulting spots were indicative of cytokine-producing T cells and were enumerated using an automated ELISpot reader (Autimmun Diagnostika gmbh ELISpot Reader Classic), and the % blockade was calculated upon the comparison of the antibody-treated and untreated groups following subtraction of the EV background level of cytokine production spots. The EV-K562 contribution (with and without antibody) was subtracted from the CD1a IFNγ spot number (with and without antibody, respectively). The adjusted CD1a-K562 antibody-treated group spot number was then divided by the CD1a without antibody group and used to calculate % blockade.

### CD1a-reactive T-cell generation and activation analysis

CD1a-restricted T cells were isolated by fluorescence-activated cell sorting. Blood T cells were co-cultured with EV-K562 of CD1a-K562 and cytokine-producing responder T cells were detected using Miltenyi MACS Cytokine Secretion assays following the manufacturer’s instructions. Briefly T cells were coated with anti-cytokine (IL-22 or IFNγ) antibody after 4 h culture with CD1a-K562 to detect CD1a-dependent autocrine cytokine production. The live responder cells were then single-cell sorted into a 96-well U-bottom culture plate. CD1a-restricted T cells were expanded with mixed lymphocyte reaction, and purity and CD1a-responsiveness were assessed with the above FACS-based cytokine secretion assay method using an analyzing flow cytometer. The activation of CD1a-restricted T cells was analyzed as follows. 2 × 10^5^ K562 cells were co-cultured with 1–5 × 10^5^ CD1a-autoreactive T cells for 4 h. Alternatively, cell-free CD1a-bead-bound assay was utilized to assess the T-cell activation. Purified and biotinylated CD1a was coated onto Streptavidin Magnetic Beads (Merck Millipore) and incubated with continuous rotation overnight at 4 °C. The unbound CD1a were removed by washing beads with culture medium three times on a magnet. Beads (1 × 10^5^) were co-cultured 4 h with blood CD1a-restricted T-cell lines (1–5 × 10^5^). Helper cytokines were added to the co-culture to support CD1a-dependent cytokine production. IFNγ-producing T-cell culture was supplied with IL-12 (1 ng/mL, BioLegend), IL-18 (1 ng/mL, BioLegend) and IL-2 (25 U/mL, BioLegend); and IL-22-producing T-cell culture were supplied with IL-6 (5 ng/mL, BioLegend), TNF-α (5 ng/mL, BioLegend) and IL-2 (25 U/mL, BioLegend). Additional 2.5 μg/ml anti-CD11a (HI111, BioLegend) was added to the bead-bound system. Activation of T cells was assessed by cytokine production of T cells using the above secretion assay (Miltenyi Biotec) following the manufacturer’s instructions.

### Murine imiquimod and MC903 administration

Mice were lightly anaesthetized with isoflurane and 15 or 25 mg Aldara cream containing 5% imiquimod or 2nmol MC903 (2700/10, Bio-Techne) dissolved in 20 µl ethanol was applied to the dorsal and ventral sides of the ear pinae on days 0, 1, 2, 3, 4, 5 in the imiquimod prevention model (Fig. 4A) or 0, 1, 2 and 4, 5, 6, 7 in the imiquimod treatment model (Fig. 6A or 0, 1, 2, 3, 4, 5, 6 in the MC903 model (Fig. 8A)). 100 µg anti-CD1a antibodies or mouse IgG1 isotype control were administered intraperitoneally on days −5, −3, −1, 1, 3, 5 in the prevention model (Fig. 4A) or 3, 5, 7 in the treatment model (Fig. 6A) or −3, −1, 0, 1, 3, 5 in the MC903 model (Fig. 8A). Ear thickness measurements were taken daily throughout the duration of Aldara/MC903 application days 0–6 in the imiquimod prevention model (Fig. 4A) or 0–8 in the imiquimod treatment model (Fig. 6A) or 0–7 in the MC903 model (Fig. 8A) using a micrometre (Mitutoyo). Twenty-four hours after the final imiquimod application, mice were euthanised by rising CO_2_ exposure and confirmed by cervical dislocation and tissues taken for analysis. Control non-imiquimod/MC903 treated mice were housed separately from topically challenged mice, as grooming behaviours could compromise untreated murine skin.

### Tissue processing

Ears, cervical lymph nodes (cLN), blood and spleen, and gut and lung tissue were collected for immunophenotyping or imaging. Cell suspensions of spleen and cLN, were obtained by passing the tissues through a 70 μm strainer and washed with RPMI containing 10% FCS. Spleen cell suspension red blood cells were removed by incubation with RBC lysis solution (eBioscience). Blood samples were collected and plasma separated by centrifugation (5 min 1000 × *g*), plasma was removed and stored (−80 °C), and cells were subjected to RBC lysis (eBioscience).

Ear skin tissue was washed in HBSS to remove excess Aldara, split ventrally, diced into <0.5 mm pieces and digested with 1 ml RPMI containing 10% FCS and 1 mg/ml Collagenase P (Roche Diagnostic) for an initial 1 hr at 37 °C. The digested tissues were briefly spun at 600 × *g* for 5 min and 100 μl of clear supernatant was collected and stored at −80 °C for LegendPlex analysis. The remaining tissue pellets were resuspended thoroughly with another 1 ml of Collagenase P digest medium for a further 1 hr incubation at 37 °C. 100 µg/ml DNase I (Roche Diagnostic) was added for a final further 30 min digestion. The digested samples were passed through 70 μm cell strainers (BD Biosciences) and washed with cold 10 mM EDTA in PBS, and a single-cell suspension was obtained in FACS cell staining buffer (PBS 10% FCS).

For the preparation of bone marrow, the femur and tibia were collected and marrow flushed with RPMI containing 10% FCS, bone marrow cells were passed through 70 μm cell strainers (BD Biosciences), and RBC lysis performed and cells were washed, and a single-cell suspension was obtained in RPMI containing 10% FCS. Bone-marrow-derived monocytes were isolated from murine bone marrow cell suspension using magnetic-activated cell sorting (CD115 Microbeads Miltenyi Biotech.). Total bone marrow cells of CD115 + bone marrow cells were cultured for 6 days in a complete medium supplemented with 20 ng/ml rmGM-CSF (BioLegend). On day 2 half of the medium was replaced and on day 3 the medium was entirely replaced, with fresh GM-CSF complete media. On day 6 non-adherent cells in the supernatant were collected and loosely adherent cells were harvested by gentle PBS washing, for separate flow cytometric analysis.

Lung tissue was disaggregated and digested with RPMI containing 10% FCS and 1 mg/ml Collagenase P (Roche Diagnostic) for 1 h at 37 °C, the partially digested tissue was passed through 70 μm cell strainers (BD Biosciences) and washed, filtered and a single-cell suspension was obtained in FACS cell staining buffer (PBS 10% FCS).

For the preparation of colonic cells, intestinal contents were removed by the application of gentle pressure along the length of the intestine. Intestines were opened longitudinally, washed in PBS and cut into ~3 mm pieces. Samples were predigested by incubation with RPMI supplemented with 10% FCS and 5 mM EDTA for 30 min at 37 °C with shaking (200 rpm). Intestinal pieces were washed with PBS/HEPES and incubated, with shaking, at 37 °C with RPMI + 2% FCS, 0.125 KU/mL DNaseI (Sigma–Aldrich) and 62.5 μg/mL Liberase TL (Roche) until no large pieces of intestine remained. Cells were then passed through a 70 μm strainer, pelleted and separated over a Lymphoprep. gradient 800 × *g* for 20 min. Colonic leukocytes were isolated from the interface and prepared for flow cytometric analysis.

#### Bead-based immunoassays

For evaluating the levels of the cytokines in the co-culture supernatant, mice skin extracts and plasma, a cytometric bead array was performed using the following LEGENDplex™ kits (BioLegend) following the manufacturer’s guidelines: Human CD8 Panel (13-plex), MU Macrophage/Microglia Panel (13-plex), MU Inflam Panel (13-plex) and MU Macrophage/Microglia Panel (13-plex). The data were acquired using LSRFortessa X-50 flow cytometer (BD Biosciences), and the analysis was performed by using LEGENDplex™ Cloud Data Analysis Software.

### Flow cytometry

For FACS surface staining the cells were incubated with TruStain FcX (1:50, anti-mouse CD16/32, 93, 101319) for 5–10 min prior to being labelled with the following anti-mouse antibodies (Biolegend sourced and used at 1:100 dilution, unless otherwise stated): CD3 (500A2, BUV495: 741064 BD Pharmingen), CD11b (M1/70, BUV395: 563553 BD Pharmingen), CD11c (N418, BV711: 117349), CD8 (53-6.7, BUV805: 612898 BD Pharmingen), CD4 (GK1.5, AF700: 100430), CD45 (2D1, FITC: 368507), CD11a (I21/7, PECy7: 153108), CD69 (H1.2F3, BV650: 104541), Langerin (4C7, PE: 144204), Ly6C (RB6-8C5, BV605: 108440), Ly6G (1A8, PETxRed: 127648), MHCII (M5/114.15.2, BV785: 107645), Siglec-F (S17007L, BV421: 155509), Live/Dead Aqua (Invitrogen), and anti-human CD1a (APC or purified SK9, HI149, OKT6, NA1/34). Gating strategies are presented in supplementary materials (Supplementary Figs. 1B, S1C, S1D, S4B, S6, S10B). Samples were analyzed on BD Fortessa X-20 or Fortessa X-50 flow cytometers, using FACS Diva v8.0 software. Data were subsequently analyzed using FlowJo v10.4.2.

### Flow cytometry: epitope competition assay

CD1a-K562 cells were incubated with purified primary newly generated and commercially available anti-CD1a antibodies on ice for 30 min (*Y*-axis Fig. 3A, 25 µg/ml), the unbound antibody was then washed away. Commercial and newly generated CD1a antibodies were conjugated with AF-647 Antibody labelling kit (Life Technologies A20186). Alexa-Fluor-647-conjugated forms of the different antibodies were then incubated with the cells on ice for 30 min (*X*-axis Fig. 3A, 10 µg/ml) in the matrix arrangement of Fig. 3A. Mean fluorescent intensity (MFI) was used to assess the degree of binding of the fluorophore-conjugated antibody.

### Confocal imaging

Murine ear skin was frozen in optimal cutting temperature embedding compound and stored at −80 °C. 10 µm cryosections were cut using a Leica cryostat and collected onto Superfrost Plus slides to air-dry for 30 min before being stored at −80 °C. Slides were rehydrated in PBS for 10 min before staining. The endogenous peroxidase activity of the sample was quenched by adding 0.15% hydrogen peroxide solution for 5 min at room temperature. Endogenous biotin was blocked with Avidin/Biotin Blocking Kit (Vector Laboratories Ltd), and 10% goat serum was used to reduce the non-specific binding of antibodies. Anti-CD1a antibody was used for confocal microscopy (1:100, OKT6; in-house production and conjugated to Biotin). Alexa Fluor 594 Tyramide SuperBoost kit (streptavidin; Thermo Fisher Scientific) was used to enhance the signal following the manufacturer’s instructions. Briefly, slides were incubated at 4 °C with primary antibodies overnight. After washing, HRP-conjugated streptavidin was added to the sections and incubated at 4 °C overnight. Excess streptavidin-HRP was washed away, the tissues were incubated with tyramide working solution for 8 min at room temperature, and the reaction was stopped with Reaction Stop Reagent. After staining, slides were mounted using antifade mounting medium with DAPI (Vector Laboratories Ltd), coverslips were applied, and slides were refrigerated in the dark until analyzed by confocal microscopy (Zeiss LSM 780 Confocal Microscope-Inverted Microscope; ×25/0.8 Imm Korr DIC M27; room temperature; Axiocam camera; BD Zeiss Zen 2011 SP7 FP3 (black) software), and (FIJI is just) Image J 1.52p was used for image processing.

### CD1a mutant protein analysis

Wild-type and mutant CD1a proteins were recombinantly produced in human embryonic kidney cells (HEK293 GnTI^−^) and purified as previously described^29^. Purified CD1a/β2m dimers were biotinylated overnight using BirA ligase. Free biotin and the enzyme were removed by a final step of gel filtration. Sample purity and biotinylation were assessed by SDS-PAGE. Biotinylated proteins (1 µg per condition) were bound to streptavidin magnetic beads (2 µl per condition) and stained with AF-647-conjugated anti-CD1a antibodies to assess binding. Commercial and newly generated CD1a antibodies were conjugated with AF-647 Antibody labelling kit (Life Technologies A20186). Data are represented as percentage MFI of AF-647 staining of CD1a wild-type binding (100%). Structural representations of epitope sites were generated with UCSF Chimera 1.15 software.

### Cytotoxicity assay

Anti-CD1a antibodies (5 µg/ml) or commercially available comparator NA1/34 were incubated with 1 × 10^5^ CD1a-expressing K562 or EV control K562 for 24–48 h and cell death assessed by flow cytometry. Briefly, some wells were harvested after 24 h culture, and to stain for dead and apoptotic cells, Zombie Fixable Viability dyes (1:1000; BioLegend) and Annexin V-APC (BioLegend) were added. To measure direct antibody-induced cell death after 48 h, K562 were fluorescently labelled with CellTraceViolet prior to incubation with anti-CD1a antibodies. To allow quantitative analysis of the surviving target cell populations, 2 × 10^5^ CFSE-labelled K562 (Reference) were added. This was done just prior to the analysis to avoid the interaction between the target, reference, and T cells. The percentage of induced killing was then calculated with the following equation by comparing the frequency of live target and reference populations: % cytotoxicity = 100−((% live CD1a-antibody-treated target cells /% live reference cells)/(% live isotype-antibody-treated target cells/% live reference cells) × 100).

### Complement-mediated lysis (CDC) and antibody-dependent cytotoxicity ADCC assays

For CDC assays, K562-CD1a cells (5 × 10^4^ cells per well) were pre-treated with either 5 µg/ml isotype control antibody or indicated antibodies for 30 min and incubated with 10% normal human serum for 3 h at 37 °C in 5% CO_2_. For ADCC assays, K562-CD1a cells (5 × 10^3^ cells per well) were co-cultured with fresh PBMCs (2.5 × 10^5^ cells per well) for 5 h at 37 °C in 5% CO_2_ with IL-2 (100U/ml) in the combination of either 5 µg/ml isotype control antibody or indicated antibodies (an effector/target ratio of 50:1). Cytotoxicity was determined by calculating the percentage of survived target K562-CD1a using the following equation: % cytotoxicity = ((% live cells of CD1a-antibody-treated CD1a-K562/% live reference K562)/(% live cells of isotype-antibody-treated CD1a-K562/% live reference K562) × 100).

### Isoelectric focusing assay (IEF)

Lipid loading was assessed by incubating 10 μg of CD1a with a 100X molar excess of imiquimod (Invivogen) solubilized in Tris Buffer saline and 2% CHAPS 7% DMSO or vehicle alone (mock) for 2 h at 37 °C and overnight at room temperature. CD1a samples were separated by isoelectric focusing (IEF). Briefly, CD1a-imiquimod and CD1a-mock proteins were loaded on an IEF pH 3-7 gel (Novex) that was then run for 1 h at 100 V, 1 h and 200 V and finally 30 min at 500 V. The gel was then fixed with 12% TCA and stained with heated SimplyBlue SafeStain for 7 min, and destained in DI water overnight.

### Statistical analysis

The one and two-way ANOVA tests or *t*-tests were performed using GraphPad Prism version 6.00 (GraphPad Software). Error bars represent the standard deviation or standard error of the mean as indicated.

### Reporting summary

Further information on research design is available in the Nature Portfolio Reporting Summary linked to this article.

## Supplementary information

Supplementary Information

Reporting Summary

## Data Availability

All data generated or analyzed during this study are included in this article and the Supplementary Information/Source Data file). Source data are provided with this paper.

## Source data

Source Data
