# Supplementary material for: CD1a promotes systemic manifestations of skin inflammation
Source: Nat Commun. 2022 Dec 7;13:7535. doi: 10.1038/s41467-022-35071-1 (PMC9729296; doi:10.1038/s41467-022-35071-1)
Supplement: Supplementary file 1 — Supplementary Information [file 41467_2022_35071_MOESM1_ESM.pdf]

Supplementary Information

CD1a promotes systemic manifestations of skin inflammation

Clare S. Hardman[1], Yi-Ling Chen[1], Marcin Wegrecki[2], Soo Weei Ng[1], Robert Murren[3], Davinderpreet Mangat[3], John-Paul Silva[3], Rebecca Munro[3], Win Yan Chan[3], Victoria O'Dowd[3], Carl Doyle[3], Prashant Mori[3], Andy Popplewell[3], Jamie Rossjohn[2,4], Daniel Lightwood[3], Graham S. Ogg[1]

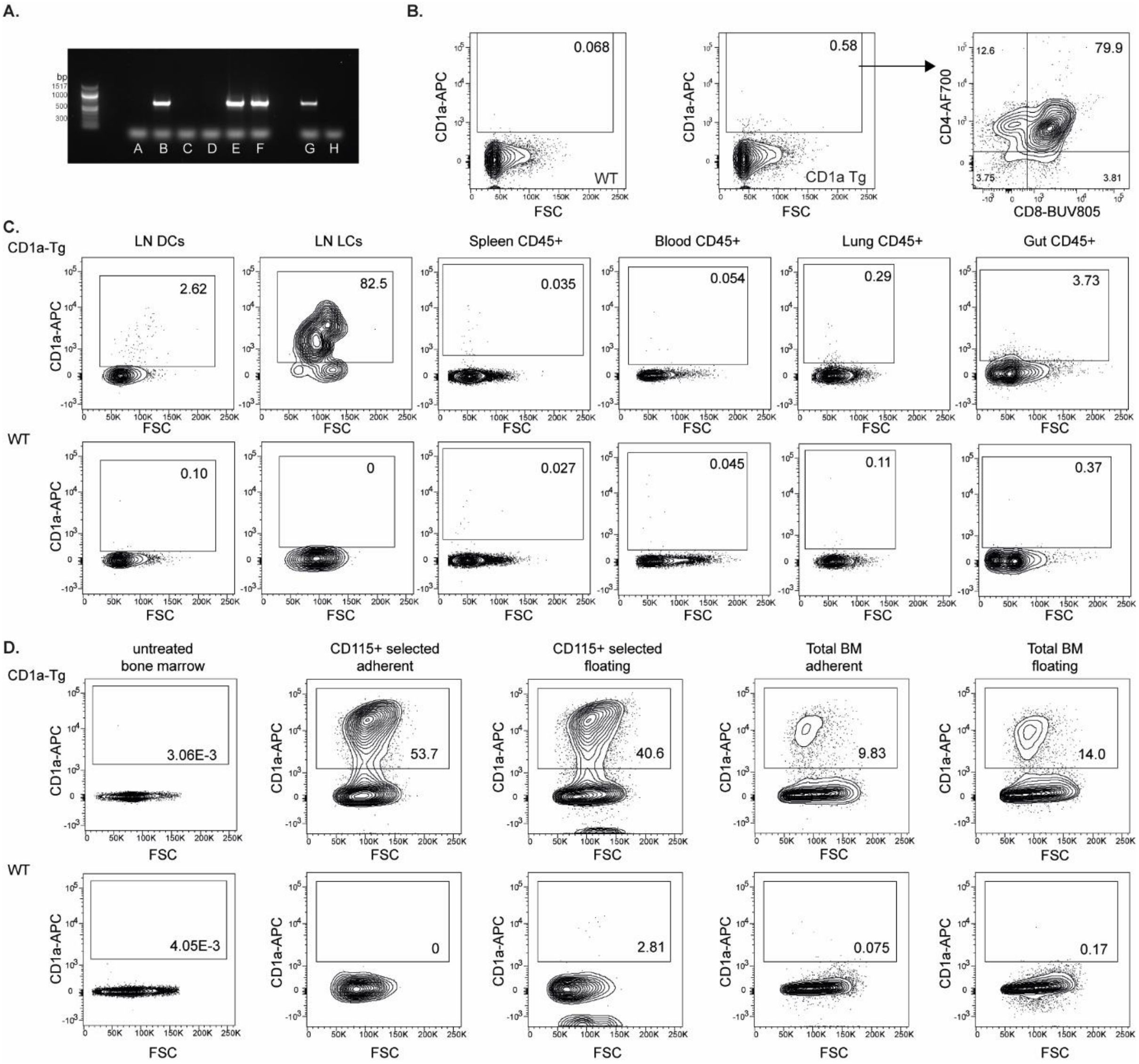

Supplementary Figure 1. Characterisation of CD1a transgenic mouse. A. Exemplar PCR genotyping of CD1a transgenic mouse line litter (lanes A-F) using CD1a forward and reverse primers and tail genomic DNA. Expected CD1a band at 655 base pairs (bp). Lane G: positive control genomic DNA from founder mouse. Lane H: negative control lacking DNA template. Data shown representative of 10 independent experiments, genotype examined once per mouse. B Representative flow cytometry plots of thymic CD1a protein expression by wild-type (WT) and CD1a transgenic (CD1a) mice. C. Steady state CD1a expression was assessed on cells derived from unchallenged naïve mice by flow cytometry. From left to right, CD1a expression was assess upon: skin draining lymph node dendritic cells (DC, CD11c+), lymph node Langerhans cells (LC, CD11c+, Langerin+), CD45+ spleen cells, CD45+ blood cells, CD45+ lung cells and CD45+ gut tissue derived cells. D. Representative flow cytometry plots of bone marrow-derived cellular CD1a protein expression by wild-type (WT, lower panels) and CD1a transgenic (CD1a, upper panels) mice. Bone marrow was collected from mice and subjected, or not, to CD115+ monocyte enrichment. Enriched and total bone marrow cells were cultured with rmGM-CSF (20ng/ml) for 7 days and CD1a expression assessed by flow cytometry of adherent and floating bone marrow derived cells. CD1a expression by untreated bone marrow was assessed directly following collection and processing.

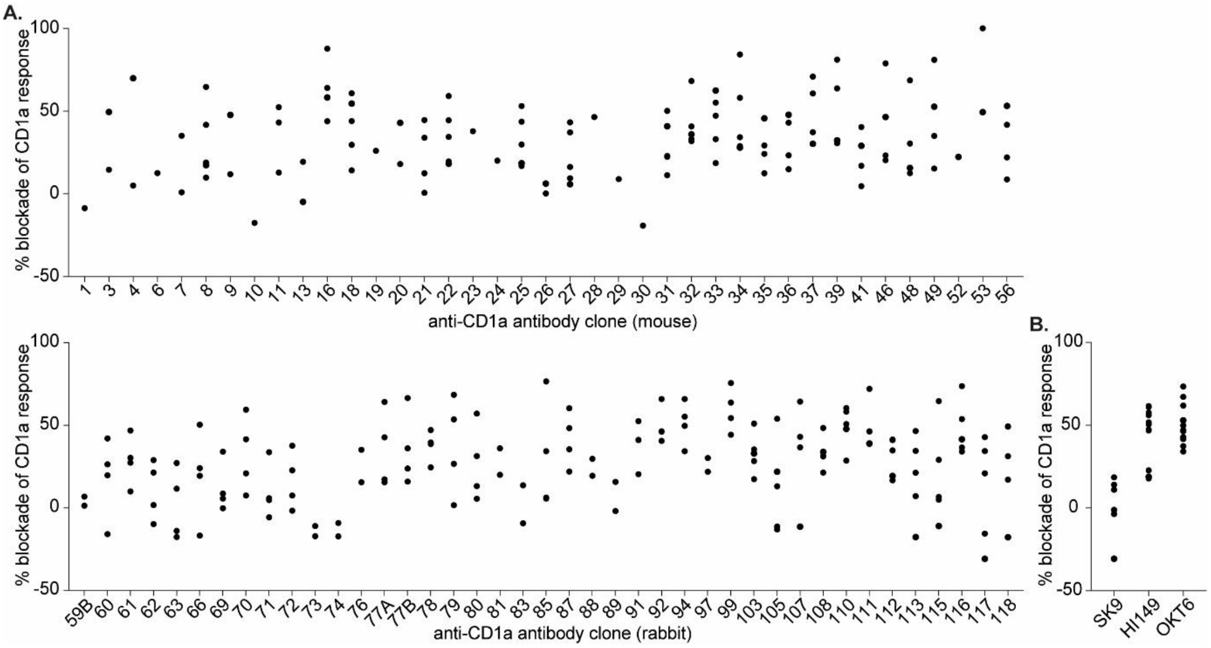

Supplementary Figure 2. Characterisation of anti-CD1a antibodies in the inhibition of polyclonal T cell responses. A-B. Determination of the capacity for a large panel of purified anti-CD1a antibodies to inhibit the CD1a dependent activation of polyclonal T cell IFNγ production. T cells were isolated from donor PBMCs by CD3 microbead separation. T cells were cocultured overnight with CD1a-K562 or EV-K562 and IFNγ production was detected by ELISpot in the presence of 10 µg / ml newly generated (**A.**) or commercially available (**B.**) anti-CD1a antibodies. % blockade was calculated upon comparison of the antibody treated and untreated groups following subtraction of the EV background level of cytokine spots. A-B. n represents biologically independent blood donors examined over 6 independent experiments. A. n=1 (1,6,10,19,23,24,28,29,30,52,53), n=2 (3,4,7,9,13,20,26,59B,73,74,76,81,83,88,89,97), n=3 (11,56) n=4 (16,21,36,37,39,41,46,49,60,61,62,63,66,69,70,71,72,77A,77B,78,79,80,85,87,91,92,94,99,107) n=5 (8,18,22,25,27,31,32,33,34,103,105,108,110,111,112,113,115,116,117,118). B. n=11 (HI149, OKT6) and n=7 (SK9). Source Data are provided as a Source Data file.

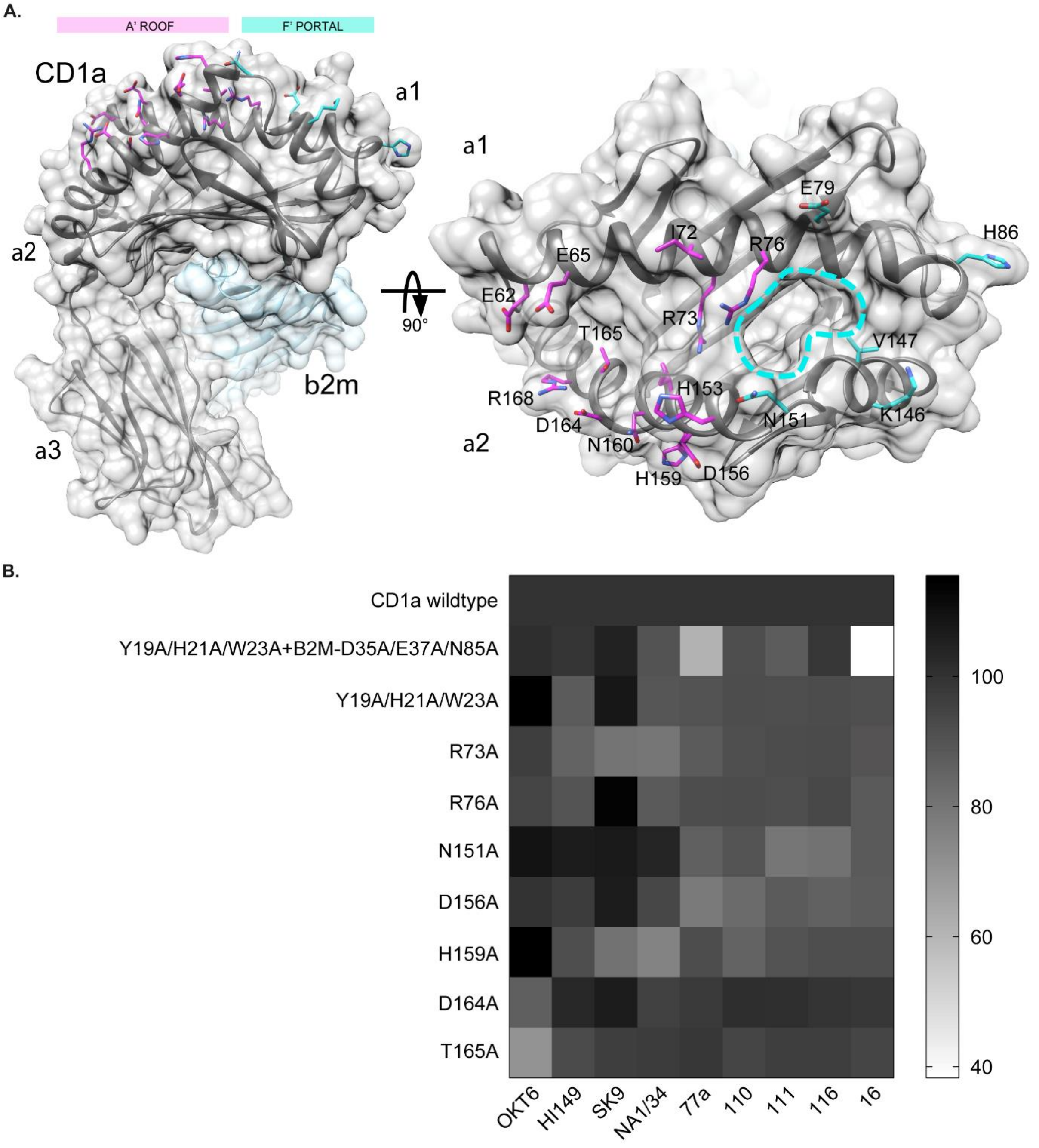

Supplementary Figure 3. CD1a antibody epitope mapping. A. Location of the mutated residues in the context of CD1a (grey) and β2m (blue) heterodimer that were used in Ab epitope mapping experiments. Corresponding α domains of CD1a are labelled. Side chains of the residues mutated to alanine are shown in colour and labelled in the right panel. Residues within the A' roof of CD1a are shown in magenta and those adjacent to the F' portal in cyan. The contour of the F' portal is marked with a cyan dashed line. **B.** Matrix heatmap representation of CD1a antibody binding to CD1a mutant proteins as represented by normalised CD1a-AF647 mean fluorescence intensity (MFI). Biotinylated CD1a mutant proteins and major and minor allele CD1a proteins were conjugated to streptavidin magnetic beads and binding of the panel of AF647-conjugated anti-CD1a antibodies was determined by flow cytometry. Grayscale shows degree of binding as a percentage of parent CD1a minor allele. Examined over 4 independent experiments.

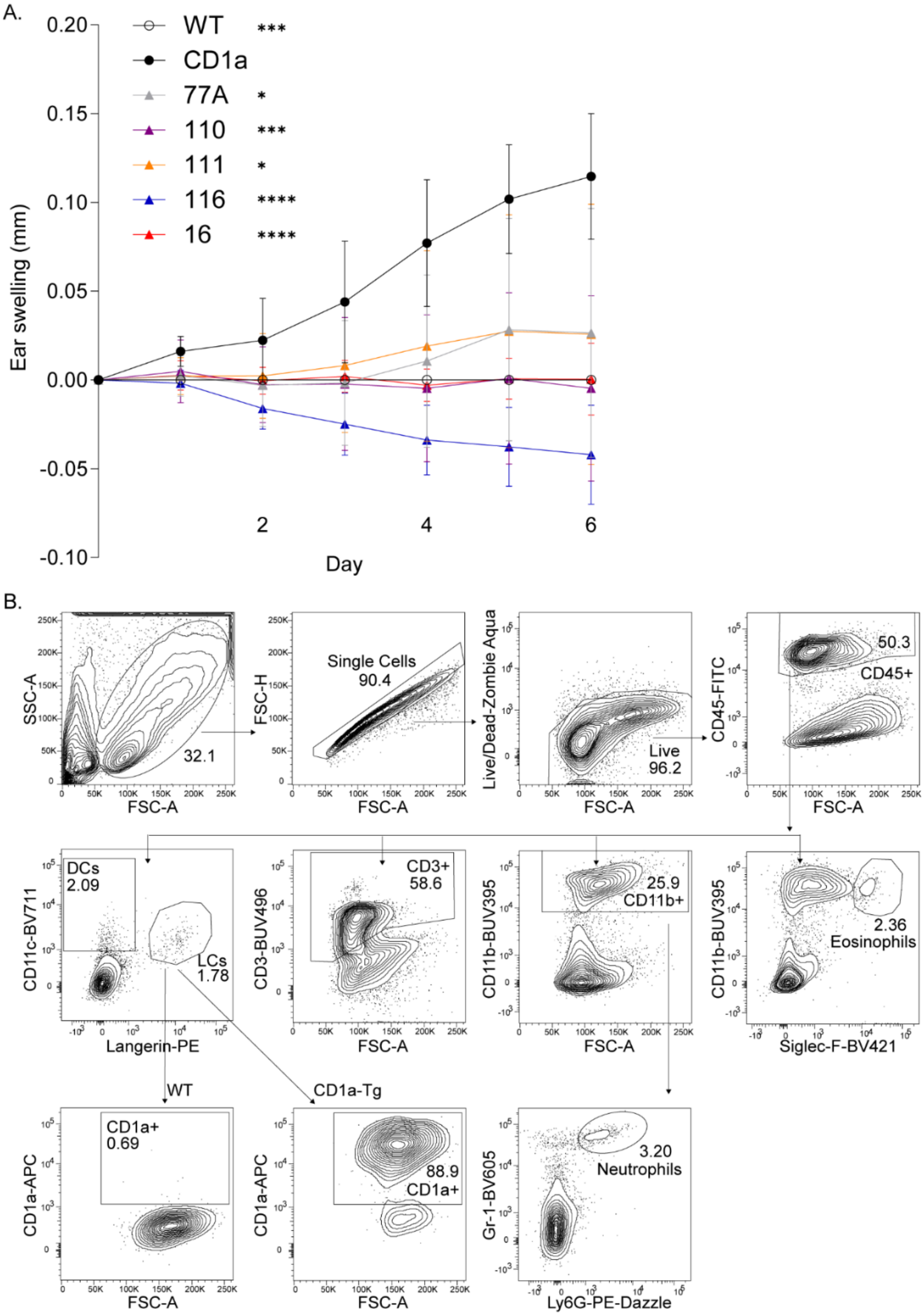

Supplementary Figure 4. Imiquimod treatment model of murine inflammation. A. Daily measurement of ear swelling induced by imiquimod treatment of wild-type (WT) and CD1a transgenic mice (CD1a) injected i.p. with mouse IgG1 isotype control and CD1a transgenic injected with the refined panel of anti-CD1a antibodies. Ear thickness was normalised to WT, mean daily WT ear thickness measurement was subtracted from the respective CD1a-Tg daily measurement. Mean ± SD is shown. n represents biologically independent animals in each group. n=6 (16) n=8 (all other groups) examined over 3 independent experiments. 2-way-ANOVA with Dunnett's test, **, P < 0.01; ****, P < 0.0001 indicates significance at day 6 on comparison to "CD1a". Exact P values: 0.0002 (WT), 0.0415 (77A), 0.0008 (110), 0.0485 (111), P<0.0001 (116, 16). Source Data are provided as a Source Data file. **B.** Exemplar gating strategies for cell types analysed *in vivo,* skin CD45+, DCs, LC, T cells, Neutrophils and Eosinophils are gated from Imiquimod treated CD1a or WT transgenic mice.

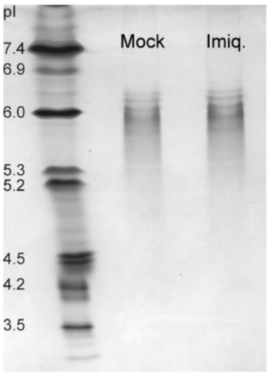

Supplementary Figure 5. Imiquimod does not constitute a CD1a ligand. Isoelectric point dependent migration of mock and imiquimod "loaded" CD1a protein on isoelectric focusing (IEF) gel pH3-7, isoelectric point (pI). Mock: vehicle control TBS 2% CHAPS 7% DMSO. Data shown representative of 2 independent experiments.

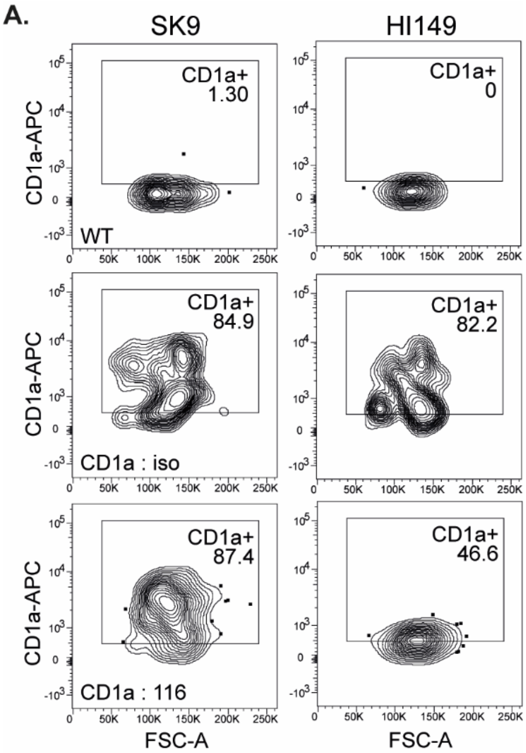

Supplementary Figure 6. *In vivo* CD1a antibody epitope competition. A. Flow cytometry plots of CD1a expression as measured by staining with anti-CD1a antibodies SK9 (left panels) or HI149 (right panels). Anti-CD1a antibody OX116 (100µg i.p.) was administered on days 0, 2 and 4 and ear skin tissue collected, processed and stained for CD1a on day 5.

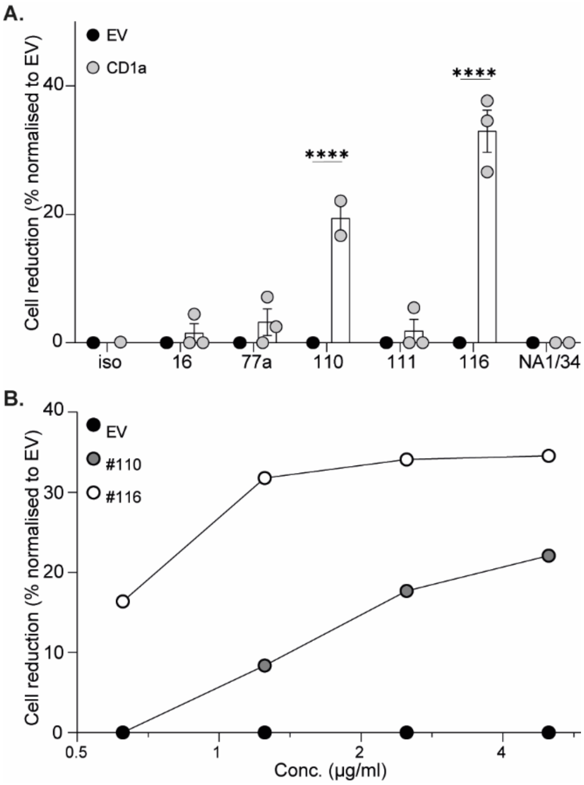

Supplementary Figure 7. Analysis of anti-CD1a antibody induced phenotype change and cytotoxicity. A. Flow cytometric analysis of antibody induced CD1a dependent phenotype change and cell reduction. Anti-CD1a antibodies or mouse IgG1 isotype control (iso, 5µg/ml) were incubated with EV or CD1a-K562 as indicated for 48 hours and percentage of antibody induced cell reduction was calculated in relation to a reference population of untreated K562 and was normalised to EV control cells (mean ± SEM). **B.** Dose titration curve of antibody induced CD1a-K562 cell reduction with increasing concentration of anti-CD1a antibody (0.625-5µg/ml). Mean ± SEM is shown. n represents independent cultures of K562 cells. n=3 (isotype, 16, 77A, 111, 116) n=2 (110, NA1/34), examined over 2 independent experiments. grouped analysis Multiple two-tailed T tests with Holm-Sidak correction, mean ± SD, ****P < 0.0001. Exact p-values are: >0.999 (iso, NA1/34), 0.9825 (16), 0.5265 (77A), <0.0001 (110, 116), 0.9476 (111). Source Data are provided as a Source Data file.

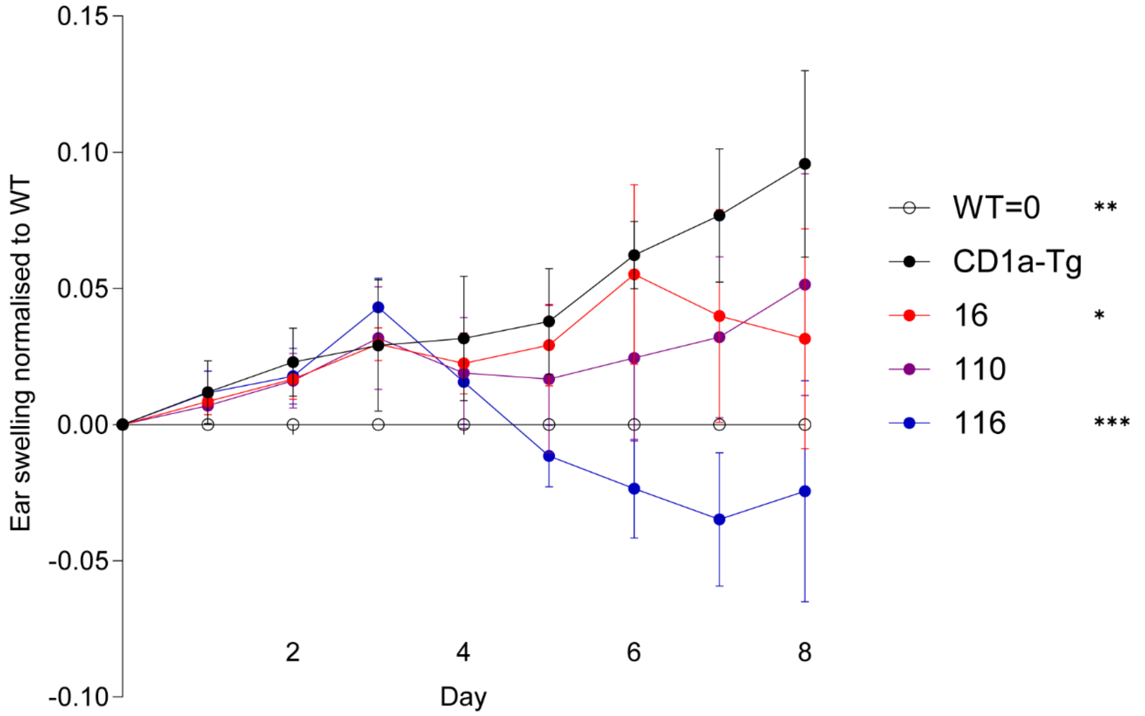

Supplementary Figure 8. Daily measurement of ear swelling induced by imiquimod treatment of wild-type (WT) and CD1a transgenic mice (CD1a) followed by the treatment i.p. with mouse IgG1 isotype control or CD1a transgenic injected with the refined panel of anti-CD1a antibodies at day 3. Ear thickness was normalised to WT, mean daily WT ear thickness measurement was subtracted from the respective CD1a-Tg daily measurement.

Mean ± SD is shown. n represents biologically independent animals in each group. B. n=4 (WT), n=6 (CD1a), n=8 (16), n=8 (110), n=10 (116), n=2 (WT unchallenged), n=6 (CD1a unchallenged), examined over 3 independent experiments. 2-way-ANOVA with Dunnett's test, *, $P < 0.05$; **, $P < 0.01$; ***, $P < 0.001$; indicates significance at day 6 on comparison to "CD1a". Exact P values: (vs CD1a) 0.0028 (WT), 0.0229 (16), 0.1297 (110), 0.0001 (116). Source Data are provided as a Source Data file.

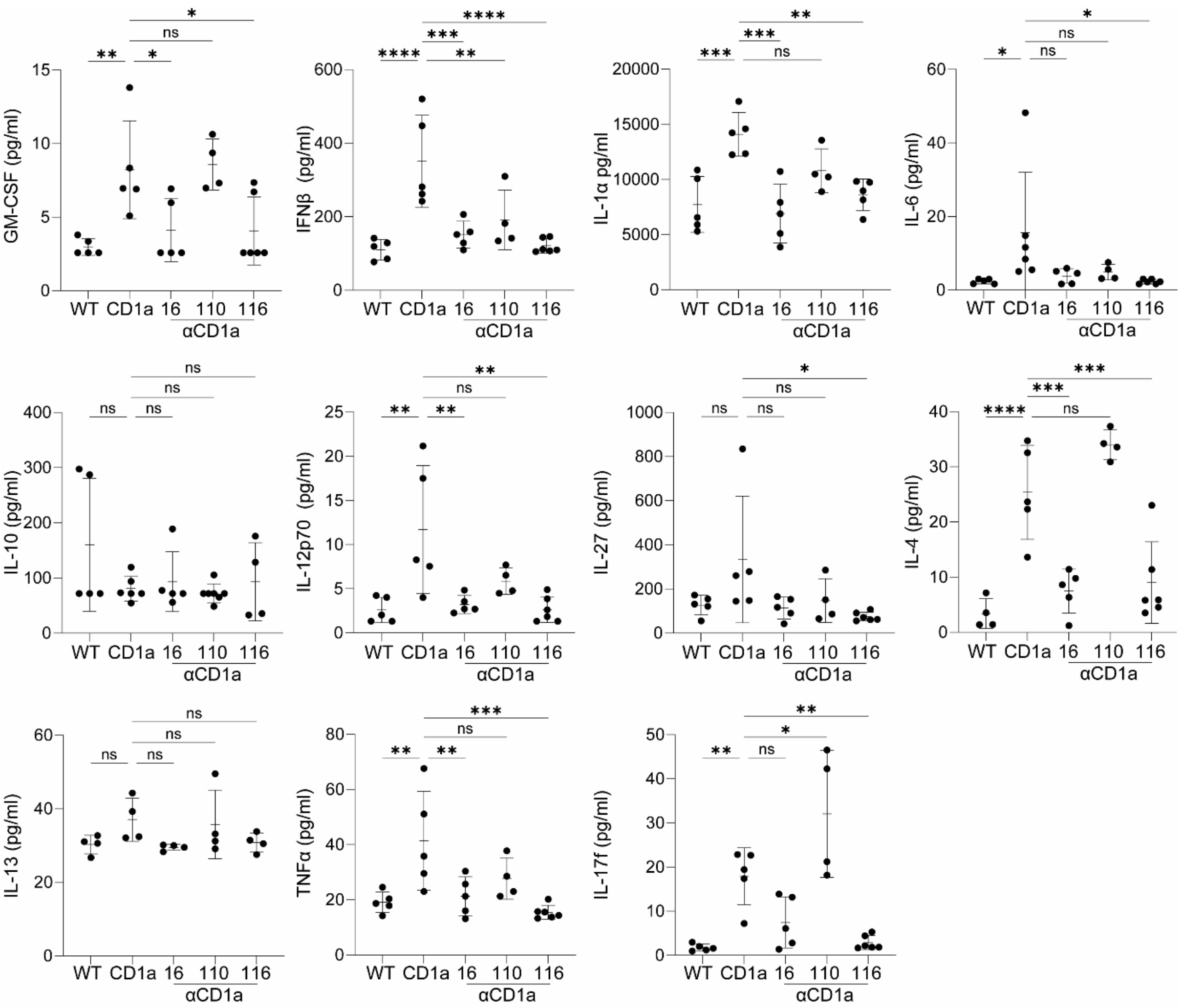

Supplementary Figure 9. Skin cytokine analysis of imiquimod-induced inflammation. Skin cytokine levels of mouse IgG1 isotype treated wild-type (WT) and CD1a transgenic (CD1a); and CD1a transgenic injected with anti-CD1a antibodies following the treatment model of administration as measured by cytometric bead array. Mean ± SD is shown. n represents biologically independent animals in each group. n=5 (WT, CD1a, 16), n=4 (110), n=6 (116), examined over 3 independent experiments. 1-way-ANOVA with Dunnett's test, *, $P < 0.05$; **, $P < 0.01$; ***, $P < 0.001$; ****, $P < 0.0001$. Exact p-values are recorded in supplementary table 8. Source Data are provided as a Source Data file.

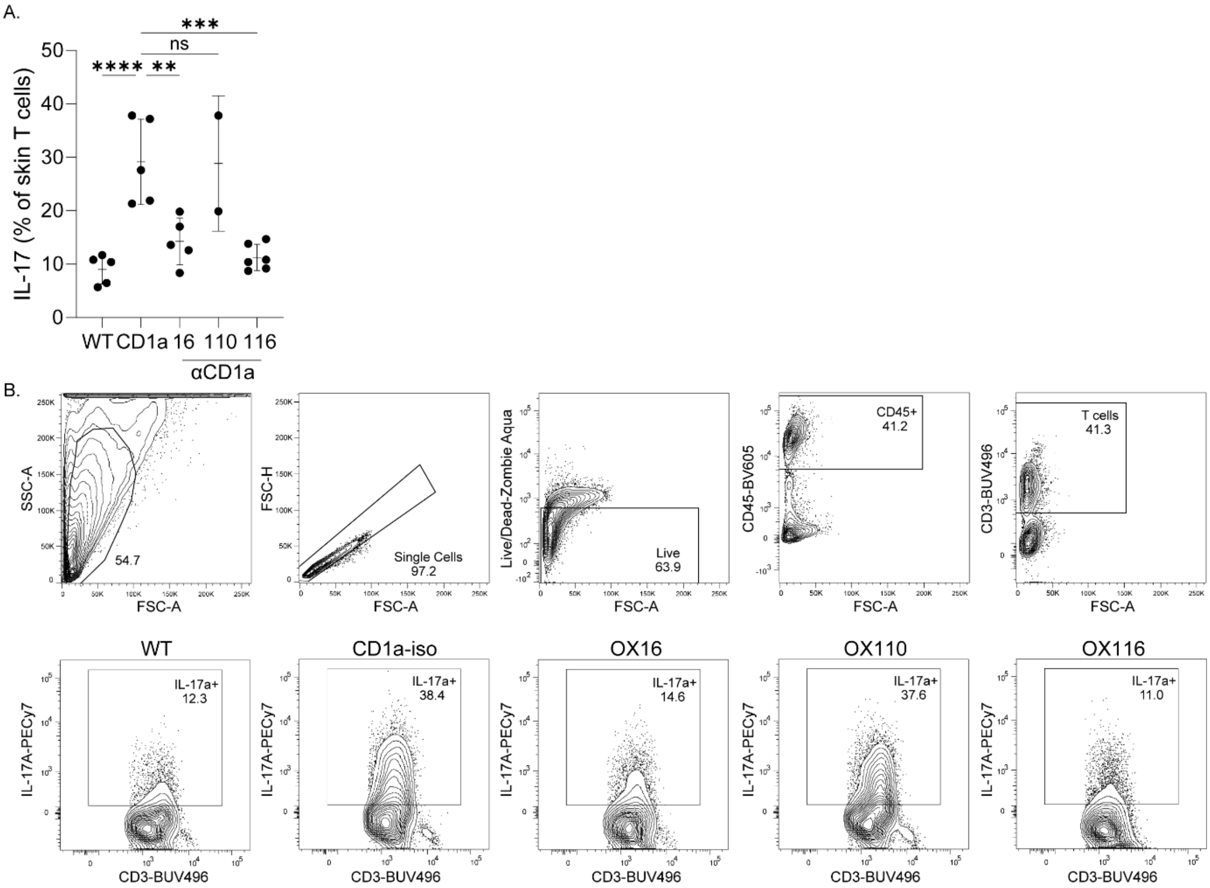

Supplementary Figure 10. Skin cytokine analysis of imiquimod-induced inflammation. **A-B.** Skin IL-17A production by CD3+ T cells of mouse IgG1 isotype treated wild-type (WT) and CD1a transgenic (CD1a); and CD1a transgenic injected with anti-CD1a antibodies following the treatment model of administration as measured by intracellular flow cytometric staining, A. graphical summary and B. representative flow cytometry plots. Mean ± SD is shown. n represents biologically independent animals in each group. n=5 (WT, CD1a, 16), n=2 (110), n=6 (116), examined over 2 independent experiments. 1-way-ANOVA with Dunnett's test, **, $P < 0.01$; ***, $P < 0.001$; ****, $P < 0.0001$. Exact p-values are: <0.0001 (WT), 0.0018 (16), 0.0002 (116), >0.9999 (110). Source Data are provided as a Source Data file.

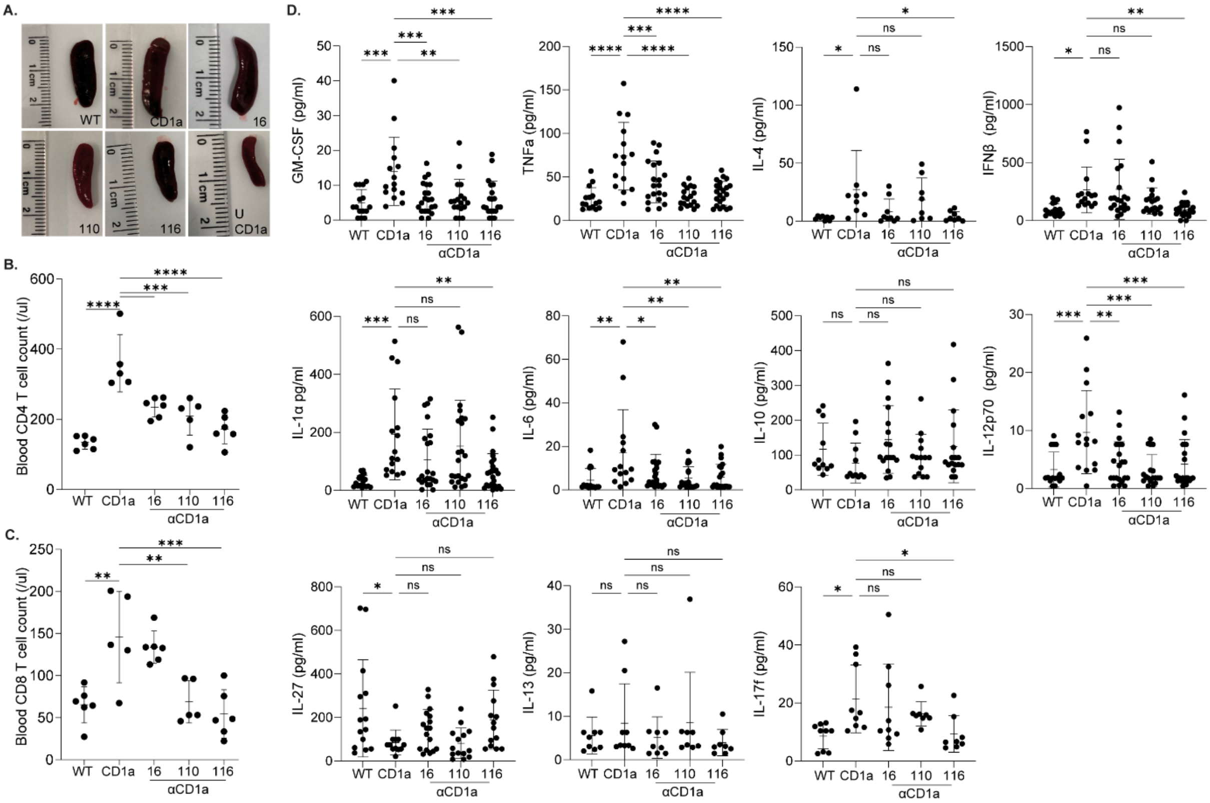

Supplementary Figure 11. Investigation of the CD1a dependency of the systemic effects of imiquimod application. A. Spleen representative images on day 8 by imiquimod treatment of wild-type (WT) and CD1a transgenic mice (CD1a) followed by treatment i.p. with mouse IgG1 isotype control or CD1a transgenic injected with the refined panel of anti-CD1a antibodies as in the schematic (Fig. 8A). **B-C.** Blood cellular analysis of the blood of mouse IgG1 isotype treated wild-type (WT) and CD1a transgenic (CD1a); and CD1a transgenic injected with the refined panel of anti-CD1a antibodies following the treatment model of administration. Circulating CD4+ (B.) and CD8+(C.) T cells were enumerated. **D.** Plasma cytokine levels of the blood of mouse IgG1 isotype treated wild-type (WT) and CD1a transgenic (CD1a); and CD1a transgenic injected with anti-CD1a antibodies following the treatment model of administration as measured by cytometric bead array. Mean ± SD is shown. n represents biologically independent animals in each group. 1-way-ANOVA with Dunnett's test, *, $P < 0.05$; **, $P < 0.01$; ***, $P < 0.001$; ****, $P < 0.0001$. B-C. examined over 3 independent experiments. D. examined over 3 (IFNβ, IL-1α, IL-6, IL-27, IL-13, IL-17f) or 4 (GM-CSF, TNFα, IL-4, IL-10, IL-12p70) independent experiments. Exact p-values are recorded in supplementary table 9. Source Data are provided as a Source Data file.

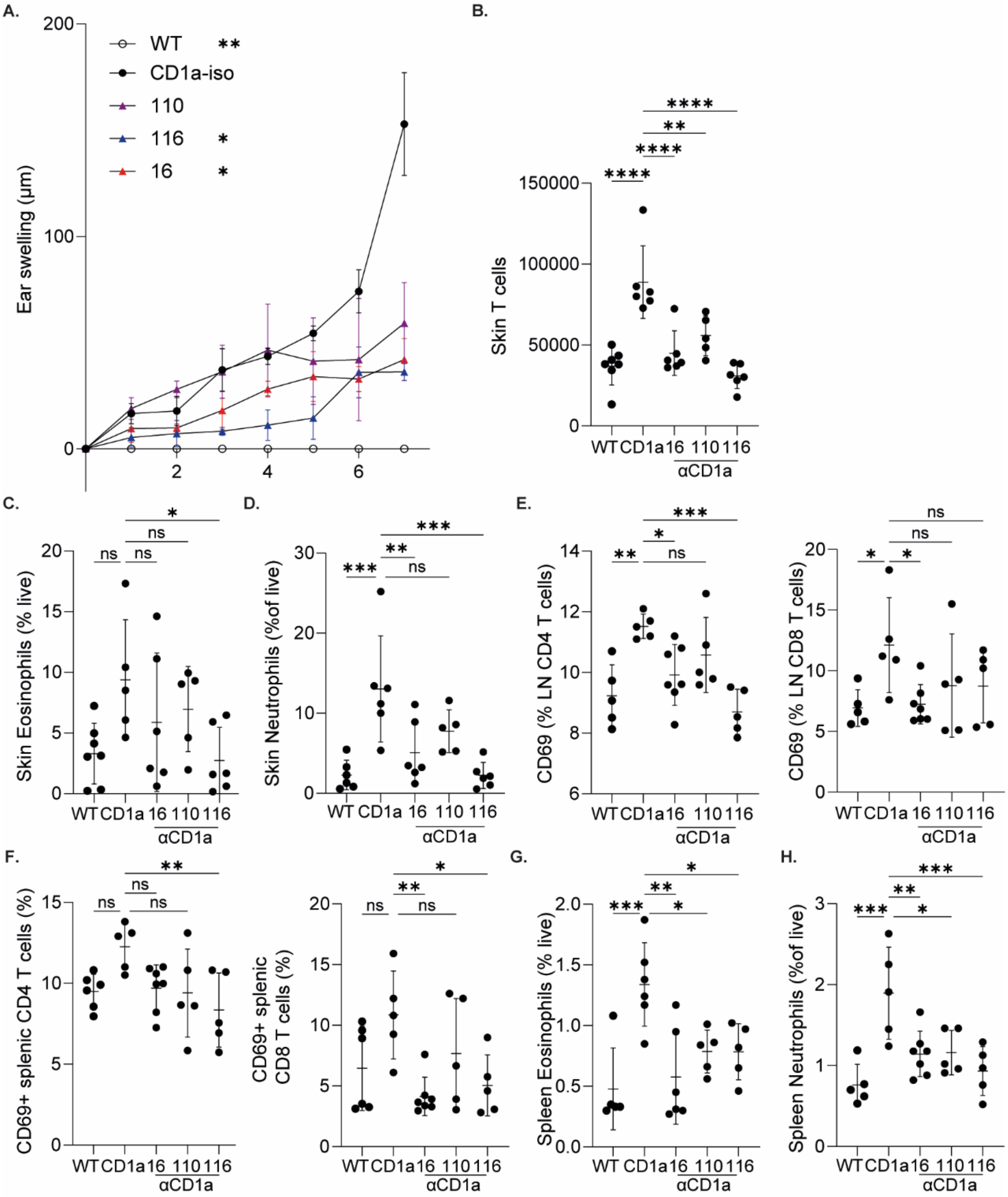

Supplementary Figure 12. Analysis of the contribution of CD1a to MC903-induced inflammation. A. Normalised daily measurement of ear swelling induced by MC903 treatment of wild-type (WT) and CD1a transgenic mice (CD1a) and countered by the treatment i.p. with mouse IgG1 isotype control or CD1a transgenic injected with anti-CD1a antibodies. Ear thickness was normalised to WT, mean daily WT ear thickness measurement was subtracted from the respective CD1a-Tg daily measurement. **B-H.** Flow cytometric analysis of immune cells derived of mouse IgG1 isotype treated wild-type (WT) and CD1a transgenic (CD1a) and CD1a transgenic injected with the refined panel of anti-CD1a antibodies. Skin T cells (/ear) (B.), eosinophils (C.) and neutrophils (D.) were enumerated, CD69 expression was assessed on CD4 and CD8 LN T cells (E.), and CD4 and CD8 splenic T cells (F.), and splenic eosinophils (G.) and neutrophils (H.) were enumerated. Mean ± SD is shown. n represents biologically independent animals in each group. A. n=5 for all groups, 2-way-ANOVA with Dunnett's test, **, P < 0.01; ****, P < 0.0001 indicates significance on comparison to "CD1a" at day 7, examined over 2 independent experiments. B-D. n=7 (WT), n=6 (CD1a, 16, 116), n=5 (110), examined over 2 independent experiments. 1-way-ANOVA with Dunnett's test, *, P < 0.05; **, P < 0.01; ***, P < 0.001; ****, P < 0.0001. E-H. n=5 (WT, CD1a, 110, 116), n=7 (16), examined over 2 independent experiments. 1-way-ANOVA with Dunnett's test, *, P < 0.05; **, P < 0.01; ***, P < 0.001; ****, P < 0.0001. Exact p-values are recorded in supplementary table 10. Source Data are provided as a Source Data file.

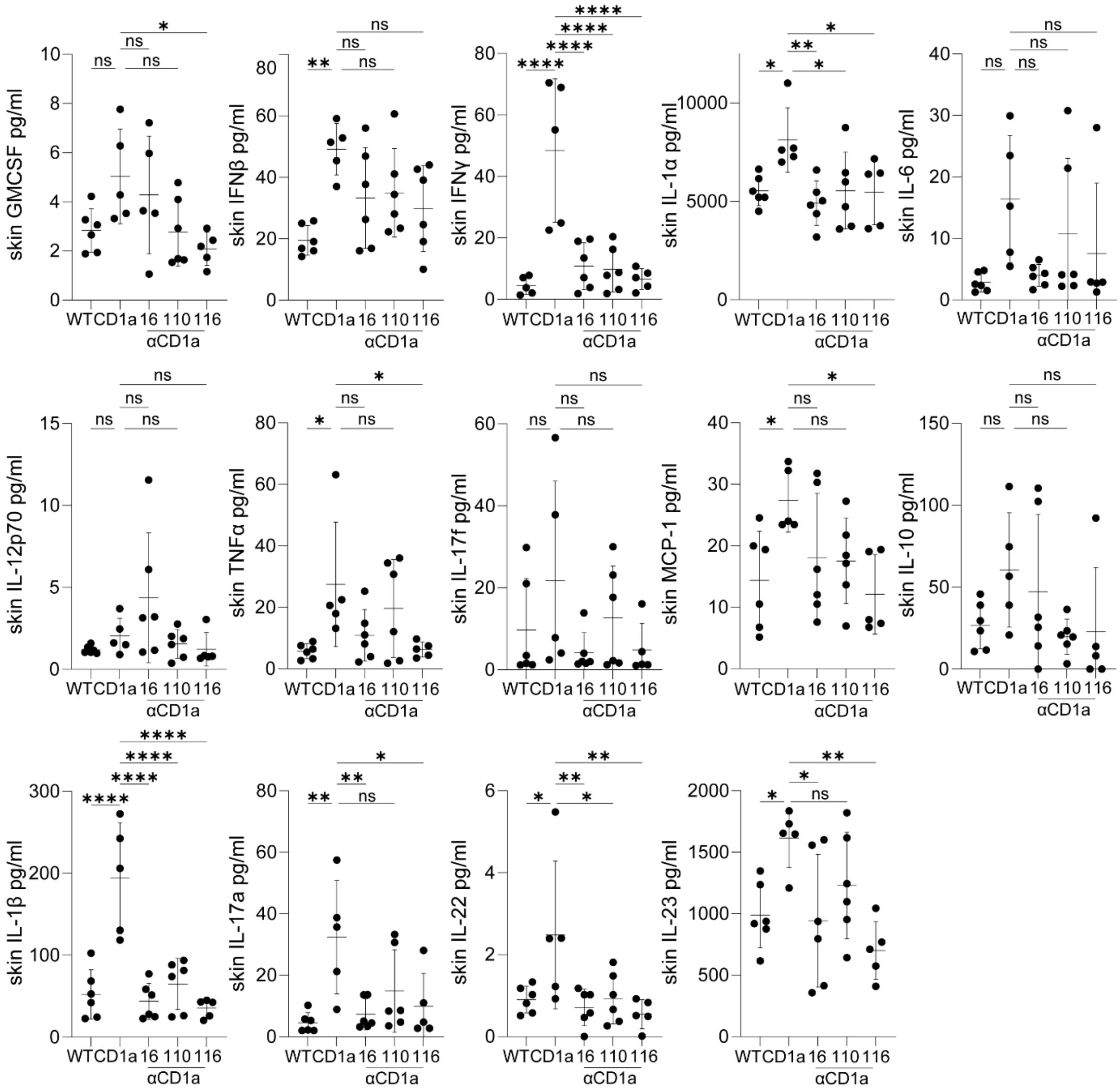

Supplementary Figure 13. Skin cytokine analysis of MC903-induced inflammation. Skin cytokine levels of mouse IgG1 isotype treated wild-type (WT) and CD1a transgenic (CD1a); and CD1a transgenic injected with anti-CD1a antibodies following the MC903 model of skin inflammation, as measured by cytometric bead. Mean ± SD is shown. n represents biologically independent animals in each group. N=6 (WT, 110), n=5 (CD1a, 116, 16), 2-way-ANOVA with Dunnett's test, *, $P < 0.05$; **, $P < 0.01$; ***, $P < 0.001$; ****, $P < 0.0001$ indicates significance on comparison to "CD1a" at day 7, examined over 2 independent experiments. Exact p-values are recorded in supplementary table 11. Source Data are provided as a Source Data file.

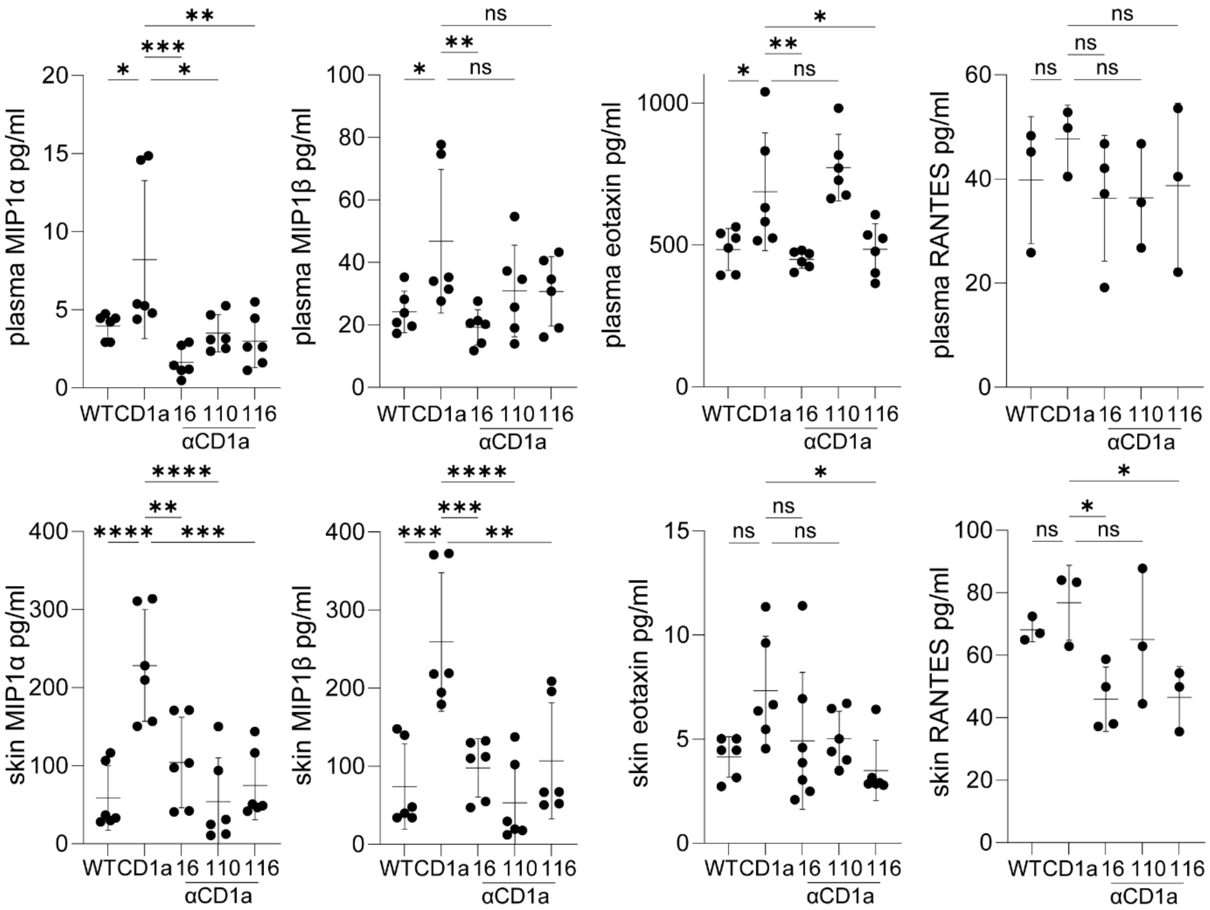

Supplementary Figure 14. Skin and plasma chemokine analysis of MC903-induced inflammation. Skin and plasma chemokine levels of mouse IgG1 isotype treated wild-type (WT) and CD1a transgenic (CD1a); and CD1a transgenic injected with anti-CD1a antibodies following the MC903 model of skin inflammation, as measured by cytometric bead array. Mean ± SD is shown. n represents biologically independent animals in each group. N=3 (RANTES), N=6 (all others), 2-way-ANOVA with Dunnett's test, *, $P < 0.05$; **, $P < 0.01$; ***, $P < 0.001$; ****, $P < 0.0001$ indicates significance on comparison to "CD1a" at day 7, examined over 2 independent experiments. Exact p-values are recorded in supplementary table 12. Source Data are provided as a Source Data file.

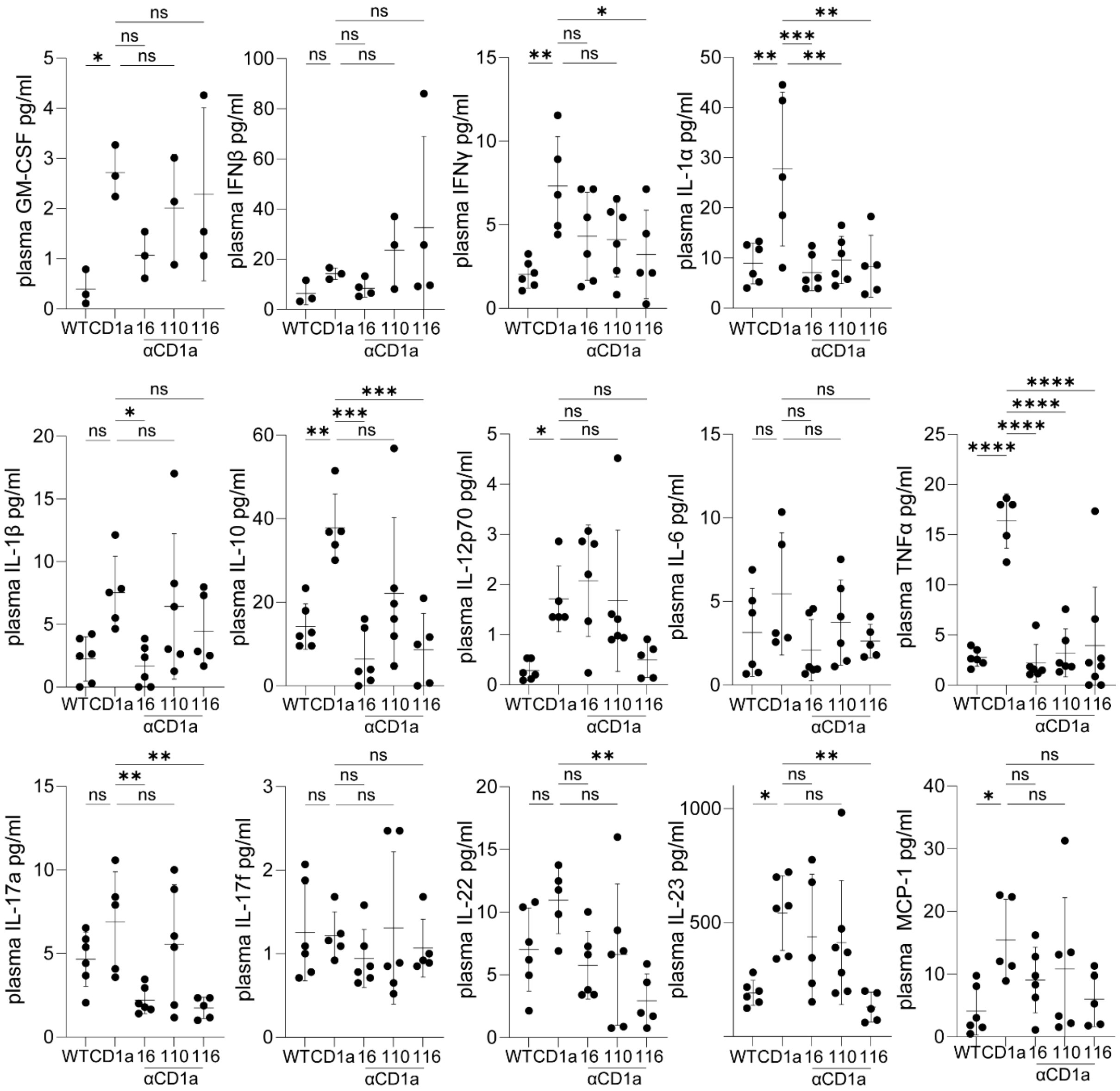

Supplementary Figure 15. Plasma cytokine analysis of MC903-induced inflammation. Plasma cytokine levels of mouse IgG1 isotype treated wild-type (WT) and CD1a transgenic (CD1a); and CD1a transgenic injected with anti-CD1a antibodies following the MC903 model of skin inflammation, as measured by cytometric bead array. Mean ± SD is shown. n represents biologically independent animals in each group. N=3 (GM-CSF, IFNb), N=6 (all others WT, 110, 16) n=5 (all others CD1a, 116), 2-way-ANOVA with Dunnett's test, *, $P < 0.05$; **, $P < 0.01$; ***, $P < 0.001$; ****, $P < 0.0001$ indicates significance on comparison to "CD1a" at day 7, examined over 2 independent experiments. Exact p-values are recorded in supplementary table 13. Source Data are provided as a Source Data file.

Supplementary Table 1. Exact P values for Figure 1.

Comparison	P value
T cells	0.030122
Eosinophils	0.000081
Neutrophils	0.0081
Langerhans cells	0.011957

Supplementary Table 2. Exact P values for Figure 2.

**Comparison**	**P value**
Fig. 2B: Dunnett's vs CD1a	
EV	0.0032
16	0.9997
34	0.9919
46	0.8561
77A	0.1495
94	0.997
99	0.9996
110	0.9938
111	0.9223
116	0.027
Fig. 2C Tukey's vs CD1a	
16	0.0327
34	0.9975
46	>0.9999
77A	>0.9999
94	0.9356
99	>0.9999
110	0.9991
111	0.9872
116	0.904
Fig. 2D Tukey's vs CD1a	
16	0.4361
34	0.871
46	0.6215
77A	0.0002
94	0.735
99	0.3943
110	<0.0001
111	0.0001
116	0.0029

Supplementary Table 3. Exact P values for Figure 4.

Comparison	P value
Fig. 4B Tukey's vs CD1a	
WT	<0.0001
16	<0.0001
77A	<0.0001
110	<0.0001
111	<0.0001
116	<0.0001
WT vs 116	0.0272
Fig. 4D Tukey's vs CD1a	
WT	0.0582
16	0.0012
77A	0.4149
110	0.0124
111	0.3282
116	0.0018
Fig. 4E Tukey's vs CD1a	
WT	0.0084
16	0.0198
77A	0.0932
110	0.1335
111	0.9994
116	0.0217
Fig. 4F Tukey's vs CD1a	
WT	0.0181
16	0.0293
77A	0.8785
110	>0.9999
111	0.8922
116	0.0003
Fig. 4G Dunnett's vs CD1a	
WT	0.0979
16	0.1723
77A	0.3465
110	00006
111	0.9914
116	0.0006
Fig. 4H Tukey's vs CD1a	
WT	0.0929
16	0.0148
77A	>0.9999
110	0.1554
111	0.0169
116	0.0009
Fig. 4I Dunnett's vs CD1a	
WT	0.0441
16	0.9463
77A	0.1197
110	0.014
111	0.8663
116	0.0007
Fig. 4J Tukey's vs CD1a	

WT	<0.0001
16	0.9892
77A	0.9998
110	<0.0001
111	0.9983
116	<0.0001
Fig. 4K Tukey's vs CD1a	
WT	<0.0001
16	>0.9999
77A	0.4647
110	<0.0001
111	0.0053
116	<0.0001

Supplementary Table 4. Exact P values for Figure 5.

**Comparison**	**P value**
Fig. 5A LC day 0 vs isotype	
16	0.6733
77A	0.9266
110	<0.0001
111	0.9997
116	<0.0001
LC day 2 vs isotype	
16	0.9674
77A	0.9988
110	<0.0001
111	>0.9999
116	<0.0001
DC day 0 vs isotype	
16	0.747
77A	0.4858
110	0.9346
111	0.9392
116	0.1529
DC day 2 vs isotype	
16	0.9999
77A	0.9999
110	0.7813
111	>0.9999
116	0.0431
Fig. 5C: EV vs CD1a	
16	0.9967
110	<0.0001
116	<0.0001
Fig. 5D: vs isotype	
16	0.9801
110	0.0003
116	<0.0001
Fig. 5E: vs isotype	
16	0.0216
110	0.0012
116	0.0006

Supplementary Table 5. Exact P values for Figure 6.

Comparison	P value
Fig. 6B Dunnett's vs CD1a	
WT	<0.0001
16	<0.0001
110	0.0003
116	<0.0001
Unchallenged WT	<0.0001
Unchallenged CD1a Tg	<0.0001
Fig. 6E Tukey's vs CD1a	
WT	0.0003
16	0.8675
110	0.0866
116	0.0011
Fig. 6E CD11a: Dunnett's vs CD1a	
WT	0.0178
16	0.9968
110	0.008
116	<0.0001
Fig. 6F Dunnett's vs CD1a	
WT	0.0016
16	>0.9999
110	0.0022
116	0.0092
Fig. 6G Dunnett's vs CD1a	
WT	0.0006
16	0.0309
110	0.0002
116	0.0006
Fig. 6H IFNγ Dunnett's vs CD1a	
WT	<0.0001
16	0.0002
110	0.0002
116	<0.0001
Fig. 6H IL-1β	
WT	0.018
16	0.0055
110	0.9999
116	0.0054
Fig. 6H MCP1	
WT	0.0016
16	0.0129
110	0.0463
116	0.0016
Fig. 6H IL-9	
WT	0.0014
16	0.0007
110	0.85
116	0.0009
Fig. 6H IL-5	
WT	0.0075
16	0.0122
110	0.0191

116	0.01
Fig. 6H IL-22	
WT	<0.0001
16	0.0003
110	>0.9999
116	<0.0001
Fig. 6H IL-17a	
WT	0.0003
16	0.0003
110	0.9694
116	0.0002
Fig. 6H IL-23	
WT	<0.0001
16	<0.0001
110	<0.0001
116	<0.0001

Supplementary Table 6. Exact P values for Figure 7.

Comparison	P value	Group size (N=)
Fig. 7A Dunnett’s vs CD1a		CD1a = 8
WT	0.0198	7
16	0.4391	9
110	0.0045	7
116	0.0002	8
Fig. 7B Dunnett’s vs CD1a		CD1a = 8
WT	0.0103	7
16	0.9999	9
110	0.2487	9
116	0.0056	9
Fig. 7C Dunnett’s vs CD1a		CD1a = 8
WT	<0.0001	7
16	0.1505	9
110	0.0002	9
116	0.0009	8
Fig. 7D Dunnett’s vs CD1a		CD1a = 8
WT	0.1585	7
16	0.3516	7
110	0.3409	8
116	0.2881	7
Fig. 7E Dunnett’s vs CD1a		CD1a = 8
WT	<0.0001	7
16	0.0313	9
110	<0.0001	9
116	<0.0001	9
Fig. 7F Dunnett’s vs CD1a		CD1a = 5
WT	<0.0001	6
16	0.0043	6
110	<0.0001	5
116	<0.0001	6
Fig. 7G Dunnett’s vs CD1a		CD1a = 5
WT	0.0008	6
16	0.0013	6
110	0.0017	5
116	0.0001	7
Fig. 7H Dunnett’s vs CD1a		CD1a = 5
WT	<0.0001	6
16	0.0032	5
110	0.002	5
116	<0.0001	7
Fig. 7I IFNγ Dunnett’s vs CD1a		CD1a = 12
WT	0.002	11
16	0.0007	14
110	0.0012	13
116	0.0008	16
Fig. 7I IL-1β		CD1a = 16
WT	<0.0001	15
16	0.3284	19
110	<0.0001	18
116	<0.0001	22
Fig. 7I MCP1		CD1a = 15

WT	<0.0001	15
16	0.0125	20
110	<0.0001	18
116	<0.0001	21
Fig. 7I IL-9		CD1a = 7
WT	<0.0001	9
16	0.0004	9
110	0.0022	8
116	0.0002	8
Fig. 7I IL-5		CD1a = 9
WT	0.0066	9
16	0.0049	9
110	0.0443	8
116	0.0184	8
Fig. 7I IL-22		CD1a = 10
WT	0.059	8
16	0.0205	8
110	0.3994	8
116	0.0236	8
Fig. 7I IL-17a		CD1a = 15
WT	<0.0001	15
16	<0.0001	23
110	<0.0001	19
116	<0.0001	20
Fig. 7I IL-23		CD1a = 15
WT	<0.0001	15
16	<0.0001	20
110	<0.0001	18
116	<0.0001	19

Supplementary Table 7. Exact P values for Figure 8.

**Comparison**	**P value**
Fig. 8B Dunnett’s vs CD1a	
WT	0.0039
16	0.0276
110	0.0717
116	0.0142
Fig. 8D Dunnett’s vs CD1a T cells	
WT	0.003
16	0.011
110	0.1337
116	0.0005
Eosinophils	
WT	0.0046
16	0.0503
110	0.3316
116	0.0024
Neutrophils	
WT	0.0015
16	0.0083
110	0.0735
116	0.0009
LCs	
WT	0.0034
16	0.0044
110	0.0105
116	0.0012
Fig. 8E Dunnett’s vs CD1a IL-4	
WT	<0.0001
16	<0.0001
110	<0.0001
116	<0.0001
Fig. 8E IL-5	
WT	0.0014
16	0.0003
110	0.0008
116	0.0002
Fig. 8E IL-9	
WT	0.0155
16	0.0074
110	0.2238
116	0.0242
Fig. 8E IL-13	
WT	0.0014
16	0.0006
110	0.0022
116	0.0018
Fig. 8F Dunnett’s vs CD1a T cells	
WT	0.0251
16	0.5874
110	0.9307
116	0.471
Fig. 8F CD4 CD11a	

WT	0.1882
16	0.007
110	0.303
116	0.0165
Fig. 8F CD8 CD11a	
WT	0.0028
16	0.0031
110	0.0079
116	0.0002
Fig. 8F eosinophils	
WT	0.0468
16	0.05
110	0.0548
116	0.0288
Fig. 8F neutrophils	
WT	0.3211
16	0.5046
110	0.146
116	0.5198
Fig. 8G Dunnett's vs CD1a IL-4	
WT	0.4004
16	0.006
110	0.1065
116	0.0026
Fig. 8E IL-5	
WT	0.0078
16	0.0004
110	0.0778
116	0.0104
Fig. 8E IL-9	
WT	0.2039
16	0.1374
110	0.9485
116	0.1396
Fig. 8E IL-13	
WT	0.0111
16	0.0131
110	0.0931
116	0.0082

Supplementary Table 8. Exact P values for Supplementary Figure 9.

Comparison	P value
GM-CSF vs CD1a	
WT	0.0049
16	0.03
110	0.9975
116	0.0205
IFNβ vs CD1a	
WT	<0.0001
16	0.0006
110	0.0083
116	<0.0001
IL-1α vs CD1a	
WT	0.0007
16	0.0002
110	0.1093
116	0.0028
IL-6 vs CD1a	
WT	0.0458
16	0.0831
110	0.1641
116	0.0335
IL-10 vs CD1a	
WT	0.16
16	0.993
110	0.9949
116	0.9978
IL-12p70 vs CD1a	
WT	0.0018
16	0.0035
110	0.0677
116	0.0013
IL-27 vs CD1a	
WT	0.088
16	0.065
110	0.1705
116	0.0192
IL-4 vs CD1a	
WT	<0.0001
16	0.0005
110	0.1317
116	0.0008
IL-13 vs CD1a	
WT	0.243
16	0.1717
110	0.99
116	0.3077
TNFα vs CD1a	
WT	0.0042
16	0.0097
110	0.1224
116	0.0007
IL-17f vs CD1a	
WT	0.0047

16	0.0813
110	0.0203
116	0.006

Supplementary Table 9. Exact P values for Supplementary Figure 11.

**Comparison**	**P value**	**Group size (N=)**
Fig. S11B Dunnett's vs CD1a		CD1a = 5
WT	<0.0001	6
16	0.0009	6
110	0.0002	110
116	<0.0001	6
Fig. S11C Dunnett's vs CD1a		CD1a = 5
WT	0.0011	6
16	0.9151	6
110	0.0028	110
116	0.0003	6
Fig. S11D GM-CSF vs CD1a		CD1a = 15
WT	0.0003	15
16	0.001	21
110	0.0014	18
116	0.0007	20
TNFα vs CD1a		CD1a = 15
WT	<0.0001	15
16	0.0006	20
110	<0.0001	18
116	<0.0001	22
IL-4 vs CD1a		CD1a = 9
WT	0.0327	8
16	0.1112	9
110	0.7257	8
116	0.0479	8
IFNβ vs CD1a		CD1a = 15
WT	0.0161	15
16	0.9999	21
110	0.256	18
116	0.0075	20
IL-1α vs CD1a		CD1a = 16
WT	0.0003	18
16	0.0632	22
110	0.6157	23
116	0.0019	25
IL-6 vs CD1a		CD1a = 14
WT	0.002	15
16	0.0236	21
110	0.0032	18
116	0.0029	23
IL-10 vs CD1a		CD1a = 11
WT	0.6194	11
16	0.1269	17
110	0.9166	14
116	0.3787	17
IL-12p70 vs CD1a		CD1a = 15
WT	0.0004	15
16	0.0019	21
110	0.0001	18
116	0.001	22
IL-27 vs CD1a		CD1a = 13

WT	0.0769	14
16	0.9641	17
110	0.896	14
116	0.4646	14
IL-13 vs CD1a		CD1a = 9
WT	0.821	9
16	0.733	9
110	>0.9999	8
116	0.5321	8
IL-17f vs CD1a		CD1a = 9
WT	0.0241	9
16	0.9237	9
110	0.6424	8
116	0.0432	8

Supplementary Table 10. Exact P values for Supplementary Figure 12.

Comparison	P value
Fig. S12A Dunnett's vs CD1a	
WT	0.0089
16	0.0516
110	0.0218
116	0.021
Fig. S12B Dunnett's vs CD1a	
WT	<0.0001
16	<0.0001
110	0.0031
116	<0.0001
Fig. S12C Dunnett's vs CD1a	
WT	0.0505
16	0.4097
110	0.7309
116	0.0369
Fig. S12D Dunnett's vs CD1a	
WT	0.0003
16	0.0055
110	0.1041
116	0.0002
Fig. S12E CD4 CD69 vs CD1a	
WT	0.0029
16	0.0262
110	0.3339
116	0.0004
Fig. S12E CD8 CD69 vs CD1a	
WT	0.0383
16	0.0345
110	0.2527
116	0.2449
Fig. S12F CD4 CD69 vs CD1a	
WT	0.0631
16	0.0778
110	0.0664
116	0.0086
Fig. S12F CD8 CD69 vs CD1a	
WT	0.0996
16	0.0055
110	0.3435
116	0.0282
Fig. S12G Dunnett's vs CD1a	
WT	0.0006
16	0.0013
110	0.027
116	0.0263
Fig. S12H Dunnett's vs CD1a	
WT	0.0002
16	0.0048
110	0.0109
116	0.001

Supplementary Table 11. Exact P values for Supplementary Figure 13.

Comparison	P value
Fig. S13 GM-CSF vs CD1a	
WT	0.0899
16	0.8539
110	0.0792
116	0.0222
IFNβ vs CD1a	
WT	0.0023
16	0.1391
110	0.2072
116	0.0567
IFNγ vs CD1a	
WT	<0.0001
16	<0.0001
110	<0.0001
116	<0.0001
IL-1α vs CD1a	
WT	0.0273
16	0.0054
110	0.0277
116	0.0307
IL-6 vs CD1a	
WT	0.0534
16	0.0843
110	0.6644
116	0.3233
IL-12p70 vs CD1a	
WT	0.8843
16	0.1909
110	0.9811
116	0.9107
TNFα vs CD1a	
WT	0.0217
16	0.0983
110	0.6493
116	0.0343
IL-17f vs CD1a	
WT	0.3982
16	0.1271
110	0.6295
116	0.1739
MCP-1 vs CD1a	
WT	0.0354
16	0.1717
110	0.139
116	0.0163
IL-10 vs CD1a	
WT	0.2612
16	0.8945
110	0.14
116	0.2147
IL-1β vs CD1a	

WT	<0.0001
16	<0.0001
110	<0.0001
116	<0.0001
IL-17a vs CD1a	
WT	0.0016
16	0.0042
110	0.0535
116	0.0145
IL-22 vs CD1a	
WT	0.02
16	0.0085
110	0.0226
116	0.006
IL-23 vs CD1a	
WT	0.035
16	0.0226
110	0.2752
116	0.0027

Supplementary Table 12. Exact P values for Supplementary Figure 14.

Comparison	P value
Plasma MIP1α vs CD1a	
WT	0.0236
16	0.0005
110	0.0114
116	0.0048
Plasma MIP1β vs CD1a	
WT	0.029
16	0.0068
110	0.1671
116	0.1616
Plasma eotaxin vs CD1a	
WT	0.0236
16	0.007
110	0.5488
116	0.024
Plasma RANTES vs CD1a	
WT	0.8202
16	0.5388
110	0.5942
116	0.7523
Skin MIP1α vs CD1a	
WT	<0.0001
16	0.0024
110	<0.0001
116	0.0002
Skin MIP1β vs CD1a	
WT	0.0002
16	0.0007
110	<0.0001
116	0.0014
Skin eotaxin vs CD1a	
WT	0.0564
16	0.1678
110	0.2195
116	0.0176
Skin RANTES vs CD1a	
WT	0.8162
16	0.0277
110	0.6269
116	0.0438

Supplementary Table 13. Exact P values for Supplementary Figure 15.

Comparison	P value
Fig. S15 GM-CSF vs CD1a	
WT	0.0473
16	0.183
110	0.7844
116	0.9486
IFNβ vs CD1a	
WT	0.9597
16	0.9825
110	0.9298
116	0.5498
IFNγ vs CD1a	
WT	0.0039
16	0.1322
110	0.101
116	0.0352
IL-1α vs CD1a	
WT	0.0018
16	0.0007
110	0.0026
116	0.002
IL-1β vs CD1a	
WT	0.055
16	0.0309
110	0.951
116	0.4231
IL-10 vs CD1a	
WT	0.0045
16	0.0003
110	0.0711
116	0.0009
IL-12p70 vs CD1a	
WT	0.0475
16	0.9029
110	0.999
116	0.1263
IL-6 vs CD1a	
WT	0.3618
16	0.1055
110	0.6067
116	0.2358
TNFα vs CD1a	
WT	<0.0001
16	<0.0001
110	<0.0001
116	<0.0001
IL-17a vs CD1a	
WT	0.3042
16	0.0078
110	0.7139
116	0.0051
IL-17f vs CD1a	

WT	0.9998
16	0.8254
110	0.9961
116	0.9781
IL-22 vs CD1a	
WT	0.2344
16	0.0793
110	0.1691
116	0.0063
IL-23 vs CD1a	
WT	0.0168
16	0.7908
110	0.5879
116	0.0068
MCP-1 vs CD1a	
WT	0.0418
16	0.3711
110	0.6372
116	0.1273

Supplementary table 14. Genotyping PCR reaction

DNA template	1ul
MyTaqRed Mix (BioLine) 2x	25ul
CD1a forward primer	0.4µM
CD1a reverse primer	0.4µM
dd-H20	Up to 50ul reaction volume

Supplementary table 15. Genotyping PCR thermocycler programme

1. Initial denaturation	95˚C, 2 min
Cycle: Steps 2-4	X34
2. Denaturation	95˚C, 20 sec
3. Annealing	60˚C, 15 sec
4. Extension	72˚C, 20 sec
5. Final extension	72˚C, 2 mins
6. Hold	4˚C
