# Supplementary material for: CD1a promotes systemic manifestations of skin inflammation
Source: Nat Commun. 2022 Dec 7;13:7535. doi: 10.1038/s41467-022-35071-1 (PMC9729296; doi:10.1038/s41467-022-35071-1)
Supplement: Supplementary file 2 — Reporting Summary [file 41467_2022_35071_MOESM2_ESM.pdf]

nature portfolio

Corresponding author(s): Graham Ogg

Last updated by author(s): 10/10/2022

Reporting Summary

Nature Portfolio wishes to improve the reproducibility of the work that we publish. This form provides structure for consistency and transparency in reporting. For further information on Nature Portfolio policies, see our Editorial Policies and the Editorial Policy Checklist.

Statistics

For all statistical analyses, confirm that the following items are present in the figure legend, table legend, main text, or Methods section.

n/a	Confirmed	
☐	☒	The exact sample size (n) for each experimental group/condition, given as a discrete number and unit of measurement
☐	☒	A statement on whether measurements were taken from distinct samples or whether the same sample was measured repeatedly
☐	☒	The statistical test(s) used AND whether they are one- or two-sided<br>*Only common tests should be described solely by name; describe more complex techniques in the Methods section.*
☒	☐	A description of all covariates tested
☐	☒	A description of any assumptions or corrections, such as tests of normality and adjustment for multiple comparisons
☐	☒	A full description of the statistical parameters including central tendency (e.g. means) or other basic estimates (e.g. regression coefficient) AND variation (e.g. standard deviation) or associated estimates of uncertainty (e.g. confidence intervals)
☐	☒	For null hypothesis testing, the test statistic (e.g. F, t, r) with confidence intervals, effect sizes, degrees of freedom and P value noted<br>*Give P values as exact values whenever suitable.*
☒	☐	For Bayesian analysis, information on the choice of priors and Markov chain Monte Carlo settings
☒	☐	For hierarchical and complex designs, identification of the appropriate level for tests and full reporting of outcomes
☒	☐	Estimates of effect sizes (e.g. Cohen's d, Pearson's r), indicating how they were calculated

Our web collection on statistics for biologists contains articles on many of the points above.

Software and code

Policy information about availability of computer code

Data collection	BD Zeiss Zen 2011 SP7 FP3 (black), FACS Diva v8.0
Data analysis	FlowJo v10.4.2, GraphPad Prism version 6.00, (FIJI is just) Image J 1.52p, LEGENDplex™ Data Analysis Software Cloud Suite, UCSF Chimera 1.15

For manuscripts utilizing custom algorithms or software that are central to the research but not yet described in published literature, software must be made available to editors and reviewers. We strongly encourage code deposition in a community repository (e.g. GitHub). See the Nature Portfolio guidelines for submitting code & software for further information.

Data

Policy information about availability of data

All manuscripts must include a data availability statement. This statement should provide the following information, where applicable:

- Accession codes, unique identifiers, or web links for publicly available datasets
- A description of any restrictions on data availability
- For clinical datasets or third party data, please ensure that the statement adheres to our policy

All data generated and analysed in this study are included in the article and supplementary files. Source data will be provided.

Field-specific reporting

Please select the one below that is the best fit for your research. If you are not sure, read the appropriate sections before making your selection.

☒ Life sciences ☐ Behavioural & social sciences ☐ Ecological, evolutionary & environmental sciences

For a reference copy of the document with all sections, see nature.com/documents/nr-reporting-summary-flat.pdf

Life sciences study design

All studies must disclose on these points even when the disclosure is negative.

Sample size	Sample size was determined based on our previous studies and those in the literature (Kim et al Nat Immunol. 2016 Oct; 17(10): 1159–1166) and is indicated in the legend.<br>Where appropriate following previous experience, in experiments designed to test whether a particular manipulation leads to a reduction in the severity of disease compared to the control group, the number of animals in each group has been calculated to ensure a 90% chance of detecting a change that is statistically significant at the 5% level. Power analysis was performed for research output including ear thickness induced by imiquimod application where for example ear thickness increase reached 0.2mm in the CD1a transgenic group and where we would like to be able to reliably detect a reduction in the wildtype group to 0.09mm, power analysis indicates a minimum group size of n=4. However other read outs including cytokine production required larger sample sizes to capture variation and expression changes for the measurement tested and the sample sizes are chosen because they provide sufficient confidence to assess the experimental results.
Data exclusions	No data were excluded from the analyses
Replication	The majority of experiments were repeated at least 3 times with independent donors/mice, as noted in the figure legends, with few being performed twice. All attempts at replication were successful, individual data points shown.
Randomization	Comparison of wild-type and CD1a-transgenic mice could not be fully randomized. Additionally due to cage grooming effects, it is not possible to randomise topical treatments within cages. Allocation of samples to the different experimental groups was based on mouse genotyping results. As part of the experimental design, biological samples from different genotypes were tested on any experimental day (where feasible) in order to avoid any technical confounders. In addition, all biological samples from different experimental groups were subject to the same protocols and treatment.
Blinding	Flow cytometry data were collected objectively using automated plate readers to avoid bias. Human participants were recruited sequentially; blinding was not required as there was no intervention.<br>No blinding was possible in the mouse studies, because experiments required repeated injections of different antibodies. A micrometer was used to empirically measure/quantify inflammation (ear thickness) rather than relying on a scoring system.<br>As allocation of samples to the different experimental groups required assessment of mouse genotyping results - blinding was not possible. However, samples from different experimental groups were processed on each experimental day. All samples were subject to same treatment and protocol.

Reporting for specific materials, systems and methods

We require information from authors about some types of materials, experimental systems and methods used in many studies. Here, indicate whether each material, system or method listed is relevant to your study. If you are not sure if a list item applies to your research, read the appropriate section before selecting a response.

Materials & experimental systems

n/a	Involved in the study	
☐	☒	Antibodies
☐	☒	Eukaryotic cell lines
☒	☐	Palaeontology and archaeology
☐	☒	Animals and other organisms
☐	☒	Human research participants
☒	☐	Clinical data
☒	☐	Dual use research of concern

Methods

n/a	Involved in the study	
☒	☐	ChIP-seq
☐	☒	Flow cytometry
☒	☐	MRI-based neuroimaging

Antibodies

Antibodies used	For FACS surface staining the cells were labelled with the following anti-mouse antibodies (Biolegend sourced unless otherwise stated): CD3 (500A2, BUV495: 741064 BD Pharmingen), CD11b (M1/70, BUV395: 563553 BD Pharmingen), CD11c (N418, BV711: 117349), CD8 (53-6.7, BUV805: 612898 BD Pharmingen), CD4 (GK1.5, AF700: 100430), CD45 (2D1, FITC: 368507), CD11a (I21/7, PECy7: 153108), CD69 (H1.2F3, BV650: 104541), Langerin (4C7, PE: 144204), Ly6C (RB6-8C5, BV605: 108440), Ly6G (1A8, PETxRed: 127648), MHCII (M5/114.15.2, BV785: 107645), SiglecF (S17007L, BV421: 155509), Live/Dead Aqua (Invitrogen), and anti-human CD1a (APC or purified SK9 (344902), HI149 (300102), OKT6 (sourced as described in reference Reinherz et al 1980 10.1073/

pnas.77.3.1588), NA1/34(ab238463, abcam)).
Newly generated OX16, OX77a, OX110, OX111, OX116 and OKT6 and commercially available SK9, HI149, NA1/34 antibodies were conjugated to Alexa-674 using AF-647 Antibody labelling kit (Life Technologies A20186).

Validation

Antibodies that are commercially available from Biolegend, Bio-Techne, eBioscience (Thermo Fisher Scientific), Abcam and Miltenyi Biotec, were validated for specificity to human or mouse antigens and for the application of flow cytometry. Validation statements are found on the manufacturer's websites:
CD3 (741064 BD Pharmingen), Application: Flow cytometry (Qualified). This antibody was developed for use in flow cytometry. The production process underwent stringent testing and validation to assure that it generates a high-quality conjugate with consistent performance and specific binding activity.
CD11b (563553 BD Pharmingen), Application: Flow cytometry (Routinely Tested)
CD11c (117349 Biolegend), Application: FC - Quality tested. Each lot of this antibody is quality control tested by immunofluorescent staining with flow cytometric analysis.
CD8 (612898 BD Pharmingen), Application: Flow cytometry (Routinely Tested)
CD4 (100430 Biolegend), Application: FC - Quality tested. Each lot of this antibody is quality control tested by immunofluorescent staining with flow cytometric analysis.
CD45 (368507 Biolegend), Application: FC - Quality tested. Each lot of this antibody is quality control tested by immunofluorescent staining with flow cytometric analysis.
CD11a (153108 Biolegend), Application: FC - Quality tested. Each lot of this antibody is quality control tested by immunofluorescent staining with flow cytometric analysis.
CD69 (104541 Biolegend), Application: FC - Quality tested. Each lot of this antibody is quality control tested by immunofluorescent staining with flow cytometric analysis.
Langerin (144204 Biolegend), Application: FC - Quality tested. IHC - Verified. Each lot of this antibody is quality control tested by immunofluorescent staining with flow cytometric analysis.
Ly6C (108440 Biolegend), Application: FC - Quality tested. Each lot of this antibody is quality control tested by immunofluorescent staining with flow cytometric analysis.
Ly6G (127648 Biolegend), Application: FC - Quality tested. Each lot of this antibody is quality control tested by immunofluorescent staining with flow cytometric analysis.
MHCII (107645 Biolegend), Application: FC - Quality tested. Each lot of this antibody is quality control tested by immunofluorescent staining with flow cytometric analysis.
SiglecF (155509 Biolegend), Application: FC - Quality tested. Each lot of this antibody is quality control tested by immunofluorescent staining with flow cytometric analysis.
CD1a SK9 (344902 Biolegend), Application: FC - Quality tested, IP, IHC-F - Reported in the literature, not verified in house. Each lot of this antibody is quality control tested by immunofluorescent staining with flow cytometric analysis.
CD1a HI149 (300102 Biolegend), Application: FC - Quality tested, IHC-F - Reported in the literature, not verified in house. Each lot of this antibody is quality control tested by immunofluorescent staining with flow cytometric analysis.
CD1a NA1/34 (ab238463, abcam)). Tested applications: Suitable for : IHC-Fr use a concentration of 1ug/ml, ICC/IF use a concentration of 1-5ug/ml

Anti-CD1a antibodies novel to this study produced by UCB-Pharma were extensively characterised as noted in the manuscript text and methods. Staining/binding was validated by ELISA and FACS using positive and negative control transfected cell lines expressing CD1a or CD1b/c/d proteins, CD1 protein coated beads and MUTZ3 cell line and primary monocyte-derived dendritic cells that endogenously express CD1a.

Eukaryotic cell lines

Policy information about cell lines

Cell line source(s)

CD1a and empty vector transfected K562 were a gift from Professor Branch Moody, Harvard Medical School, see reference de Jong et al., Nature Immunology, 2010, volume 11, 1102–1109
Expi293 were sourced from Thermo Fisher Scientific.

Authentication

The cell lines were not authenticated, other than for expression of the transfected protein (CD1a).

Mycoplasma contamination

Cell lines were routinely tested for mycoplasma and were negative

Commonly misidentified lines
(See ICLAC register)

No commonly misidentified cell lines were used

Animals and other organisms

Policy information about studies involving animals; ARRIVE guidelines recommended for reporting animal research

Laboratory animals

CD1a transgenic mice were generated by the Wellcome Trust Centre for Human Genetics, Oxford on a C57BL/6J background. Transgenic offspring were identified by PCR using transgene specific primers and bred as individual lines with wild-type C57BL/6J mice. All mice were bred in a specific pathogen-free facility. In individual experiments, hemizygous transgenic mice were matched for age (experiments performed at 6-10 weeks old), sex and background strain with wild-type litter mates/counterparts used as matched controls. Mice were maintained in a pathogen-free facility in individually ventilated cages in an ambient temperature- and humidity-controlled room with a 12 h light/12 h dark cycle under standard housing conditions with continuous access to food and water.

In the generation of anti-CD1a antibodies, female Balb/C Mice 11-13 weeks old were immunized with syngeneic cells transfected with human CD1a and mouse β2M and Female New Zealand White rabbits 12-16 weeks old were immunized with syngeneic cells transfected with human CD1a and rabbit β2M. Mice were housed in conventional cages with temperatures maintained between 19-230C and a humidity of around 55% +/- 10%. Rabbits were housed in floor pens with temperatures in the room maintained

between 16-200C with the same humidity. For both mice and rabbits. 12 hours light 12 hours dark are cycled on the system from 7 to 7.

Wild animals	The study did not involve wild animals
Field-collected samples	The study did not involve samples collected from the field
Ethics oversight	All experiments undertaken in this study were done so with the approval of the UK Home Office.

Note that full information on the approval of the study protocol must also be provided in the manuscript.

Human research participants

Policy information about studies involving human research participants

Population characteristics	Human cells were isolated from individuals of different, age (25-50), balanced gender distribution of healthy controls.
Recruitment	Blood samples were provided by healthy volunteers, from The Weatherall Institute of Molecular Medicine, or who visited the Churchill Hospital Department of Dermatology, Oxford. Samples were de-identified to limit preselection bias. Participants were recruited sequentially; blinding and randomization were not required as there was no intervention.
Ethics oversight	PBMCs were isolated from healthy adult donors under local ethics approval (National Research Ethics Service Committee South Central, Oxford C, 14/SC/0106).

Note that full information on the approval of the study protocol must also be provided in the manuscript.

Flow Cytometry

Plots

Confirm that:

☒ The axis labels state the marker and fluorochrome used (e.g. CD4-FITC).

☒ The axis scales are clearly visible. Include numbers along axes only for bottom left plot of group (a 'group' is an analysis of identical markers).

☒ All plots are contour plots with outliers or pseudocolor plots.

☒ A numerical value for number of cells or percentage (with statistics) is provided.

Methodology

Sample preparation	Human blood samples:<br>PBMCs were isolated with Lymphoprep density gradient from healthy adult donors and T cells purified using anti-CD3 magnetic bead sorting following the manufacturer's protocol (MACS, Miltenyi).<br>Mouse Tissue processing:<br>Mice were sacrificed and tissues taken 24 h after final imiquimod challenge. Ears, cervical lymph nodes (cLN) and spleen were collected for immunophenotyping or imaging. Cell suspensions of spleen and cLN, were obtained by passing the tissues through a 70 µm strainer and washed with RPMI containing 10% FCS. Spleen cell suspension and whole blood red blood cells were removed by incubation with RBC lysis solution (eBioscience).<br>Ear skin tissue was washed in HBSS to remove excess imiquimod, split ventrally, diced into <0.5mm pieces and digested with 1 mg/mL collagenase P (Roche) and 0.1 mg/mL DNaseI (Sigma-Aldrich) DMEM for 3-5x30mins with agitation, dispase 5mg/mL was added to the final 30min digest step. A single cell suspension was obtained upon washing with DMEM containing 10% FCS through a 30 µm strainer prior to analysis by flow cytometry.
Instrument	For flow cytometric analysis BD Fortessa was used.
Software	Flow Jo V10 was used for all flow cytometry analysis
Cell population abundance	Sorted CD1a restricted T cell lines constituted more than 80% CD1a restricted cells. Purity was assessed based on subsequent functional analysis in response to CD1a coated beads or CD1a expressing cells and on TCR sequencing results of paired TCR. CD1a restricted lines were selected for analysis.
Gating strategy	CD1a gating performed on CD1a transgenic mice using wildtype litter mates as a negative control for anti-CD1a antibody staining as used in figure 1A.<br>Gating strategy for CD1a+ thymocytes provided in figure S1B.<br>Full gating strategy will be shown in the supplementary files:<br>For figure 1: FSC/SSC, single cells (FSC-A/FSC-H), live cells, CD45+: CD11c+langerin- or CD11c+Langerin+, CD1a<br>For figure 2: FSC/SSC, single cells (FSC-A/FSC-H), live cells, CD3+T cells, IFNg/IL-22 secretion<br>For mouse data:<br>T cells: FSC/SSC, single cells (FSC-A/FSC-H), live cells, CD45+, CD3+, CD4 or CD8, CD69 or CD11a<br>Langerhans cells: FSC/SSC, single cells (FSC-A/FSC-H), live cells, CD45+, CD11c+, Langerin+, CD1a<br>DCs: FSC/SSC, single cells (FSC-A/FSC-H), live cells, CD45+, CD11c+, MHC+, Langerin-

Neutrophils: FSC/SSC, single cells (FSC-A/FSC-H), live cells, CD45+, CD11b+, Ly6G+, Gr1-hi
Eosinophils: FSC/SSC, single cells (FSC-A/FSC-H), live cells, CD45+, CD11b+, SiglecF+

☒ Tick this box to confirm that a figure exemplifying the gating strategy is provided in the Supplementary Information.
